# Programmable Doped Metal Oxide Nanocrystals via Continuous Growth

**DOI:** 10.1021/acsnano.6c02958

**Published:** 2026-06-06

**Authors:** Nicolò Petrini, Andrea Camellini, Andrea Rubino, Priyadarshi Ranjan, Irene Martin, Enrico Squiccimarro, Sidharth Kuriyil, Anjana Muraleedharan Panangattil, Francesco Scotognella, Nicola Curreli, Teresa Gatti, Ilka Kriegel

**Affiliations:** 1 Department of Applied Science and Technology, 19032 Politecnico di Torino, Torino 10129, Italy; 2 Nanochemistry, Istituto Italiano di Tecnologia, Genova 16163, Italy; 3 Center for Sustainable Future Technologies, Istituto Italiano di Tecnologia, Torino 10144, Italy; 4 Department of Electrical and Electronic Engineering, 3111 University of Cagliari, Via Marengo 2, Cagliari 09123, Italy; 5 Photonic Nanomaterials, Istituto Italiano di Tecnologia, Genova 16163, Italy

**Keywords:** metal oxide nanocrystals, continuous-growth synthesis, doping control, indium tin oxide, localized surface plasmon resonance, post-synthesis LSPR tunability, depletion layer, thin films of nanocrystals, light-based energy applications

## Abstract

Doped metal oxide nanocrystals have emerged as a versatile platform for optoelectronic, catalytic, and energy-related applications, owing to their tunable electronic structure, chemical robustness, and solution processability. Recent advances in continuous injection (“living”) synthesis have transformed these materials from static products of batch reactions into programmable inorganic architectures, enabling deterministic control over size, faceting, surface chemistry, and, critically, radial dopant distribution. In this Review, we examine how precursor flux, reagent identity, and temporal dopant delivery encode growth pathways that directly map onto plasmonic response, charge transport, electrochromic behavior, and chemical reactivity. We highlight how controlled dopant placement and surface electrostatics define depletion layers and tune active nanocrystal response. Beyond optical and electronic function, we discuss emerging opportunities in catalysis, photoinduced charge storage, and chromogenic devices, where nanocrystals designed at the synthesis stage enable functionalities not accessible through postsynthetic modification alone. Finally, we outline future directions toward predictive synthesis and scalable integration, positioning continuous growth as a general design framework for next-generation functional oxide-based nanomaterials.

## Graphical abstract

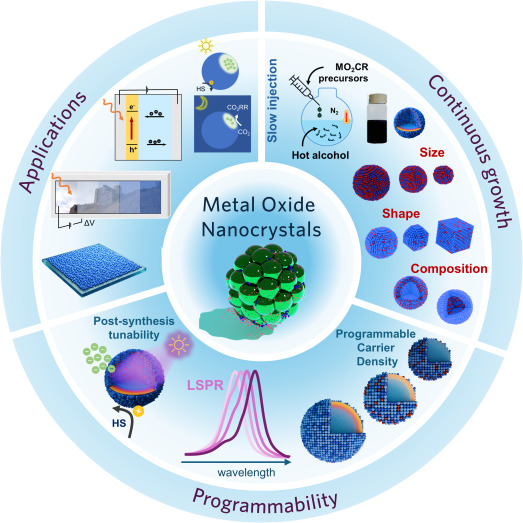

## 1 Introduction

Doped metal oxide nanocrystals (MO NCs) have emerged as a versatile materials platform whose electronic behavior can be tuned from insulating to semiconducting or even metallic through controlled lattice composition and dopant chemistry.^1–3^ This tunability has positioned doped MO NCs as promising candidates for a broad spectrum of applications in optoelectronics, from energy conversion to storage technologies.^4,5^ In these materials, interesting functional properties typically arise from aliovalent doping, which introduces free charge carriers and modifies the local electronic structure; however, the fundamental behavior of such materials also depends critically on how dopants are incorporated within the crystal lattice or rather the placement across the structure.^6–9^ At the nanoscale, this sensitivity is further magnified. High surface-to-volume ratios, quantum confinement effects, and solution processability not only expand the design space for device integration but also introduce new constraints on doped MO behavior. Well known colloidal synthesis approaches, such as hot-injection and heat-up methods, already offer specific control over nanocrystal size and morphology.^10,11^ Nevertheless, the spatial distribution of dopants remains an elusive factor in the performance paradigm. As nanocrystal size approaches fundamental length scales associated with dopant–dopant spacing, space-charge depletion regions, and surface band bending, dopant location effects become noteworthy.^7^ Dopants may segregate toward surfaces, remain electronically inactive, or contribute to the formation of depleted or insulating regions, thereby undermining the intended electronic response.^6^ Precise, spatially resolved dopant placement is therefore essential to fully exploit the intrinsic potential of doped MO NCs. These challenges have found new perspectives in the development of synthetic strategies that move beyond monodispersity and shape control toward deterministic control over dopant incorporation. In this context, recent advances in continuous-injection and continuous-growth methodologies provide a powerful framework to control MO NCs formation through a well-defined monomer supply throughout the reaction.^12–15^ By tuning precursor reactivity, injection rate, and surface chemistry, these approaches establish kinetic regimes in which faceting, anisotropic growth, and dopant incorporation can be systematically and predictively controlled.^14,15^ As a result, compositional and structural features can be programmed directly during nanocrystal growth, rather than retrofitted through postsynthetic modification. Collectively, these developments point toward a programmable synthesis. Translated to colloidal MO NCs, “programmability” does not necessarily imply postsynthesis reconfiguration but rather indicates that the synthetic pathway itself provides sufficient precision to encode a targeted nanoscale architecture and, consequently, a desired property set. Programmable functionality arises from the integrated control of composition, spatial distribution, and defect-mediated electronic structure. Achieving such control requires moving beyond conventional synthetic parameters. In addition to temperature, ligands, and precursor concentration, programmable synthesis depends on factors such as nucleation and growth mechanisms, the evolution of particle number, temporal control of precursor delivery, precursor conversion kinetics, monomer activity, dopant diffusion, and the accessibility of metastable dopant distributions. While traditional methods such as hot-injection or one-pot doping are effective for producing monodisperse particles, they often couple nucleation, growth, and dopant incorporation within narrow time windows, limiting independent control over composition and spatial distribution. Within this framework, continuous growth emerges as the most effective platform for programmable synthesis of doped MO NCs.

In this review, we examine how controlling growth pathways and dopant placement in colloidal MO NCs can reshape the ability to engineer electronic structure and function at the nanoscale. We focus on the mechanistic links between monomer speciation, flux, and surface chemistry and how these parameters steer morphological evolution while enabling spatially resolved dopant incorporation. Indium tin oxide (Sn:In_2_O_3_, ITO) nanocrystals serve as a central case study due to their rich interplay between dopant placement and activation, depletion layer formation, surface effects, and optoelectronic response and reactivity. Continuous growth enables directional control over dopant incorporation, allowing dopant placement to follow growth trajectories analogous to layered or sedimentary deposition. Such radially or axially layered architectures directly modulate the electronic landscape of the nanocrystal and enable precise tuning of optoelectronic behavior, which is directly manifested in the plasmonic response. Beyond experimental observations, substantial effort has been devoted to theoretical modeling, aimed at describing and predicting how spatially resolved dopant distributions reshape the electronic structure and drive emergent reactivity arising from gradual, nonuniform potential and carrier-density profiles. Building from this model system, we outline generalizable concepts applicable to a broad class of metal oxides and highlight emerging opportunities for predictive synthesis and the rational design of multifunctional nanocrystals.^12–14^

The review is organized as follows. Section 2 introduces the principal synthetic routes to colloidal MO NCs, emphasizing how precursor chemistry and ligand environments govern nucleation and growth. Section 3 focuses on continuous-growth (“living”) synthesis strategies, detailing the mechanistic roles of monomer generation, precursor flux, surface chemistry, and reagent identity in controlling nanocrystal size, faceting, crystallinity, and radial dopant placement. Section 4 discusses how controlled dopant incorporation and spatial dopant distributions dictate the optical response of MO NCs, with particular attention to dopant activation, plasmonic behavior, depletion-layer effects, and the modeling of their plasmonic response. Section 5 connects these programmable nanocrystals to solid-state films and applications, while also outlining emerging opportunities.

## 2 Metal Oxide Nanocrystals Synthesis

While semiconductor nanocrystals have been extensively studied over the past two decades,^16,17^ MO NCs represent a distinct and highly versatile subclass.^18^ Their relevance arises from their chemical robustness and unique optoelectronic behavior. In contrast to conventional semiconductor nanocrystals, many MO systems exhibit wide bandgaps, which generally lead to limited photoluminescence efficiency, although notable exceptions exist, such as doped ZnO.^19^ At the same time, these materials support highly tunable localized surface plasmon resonances (LSPR).^20^ Unlike metallic nanocrystals, where plasmonic properties are largely dictated by intrinsic carrier density, MO NCs systems enable broad modulation of the optical response, typically in the infrared region, through controlled aliovalent doping (e.g., ITO,^21^ ICO,^22^ AZO^19^) and defect engineering such as the introduction of oxygen vacancies (e.g., ZnO,^23^ TiO_2_,^24^ WO_3_ ^25^). The possibility of achieving higher doping levels compared to other semiconductors further enhances their appeal for electronic and plasmonic applications, while maintaining partial transparency in the visible range.^1,17,26,27^ Moreover, compositional flexibility across a wide range of oxides and dopant chemistries, enables access to diverse functionalities, including magnetic properties (paramagnetism in isovalent substituted ZnO^19,28^ and TiO_2_,^24^ ReO_3_,^29^ ferromagnetism in Fe_3_O_4_,^30^ antiferromagnetism in CoO^31^), electro and photochromism (ITO,^32,33^ ZnO,^34^ WO_3_,^25,35^ MoO_3_,^36^ ICO^37,38^ ), phase-transition electronic behavior (VO_2_,^39^ ReO_3_ ^40^). Synthetic routes to fabricate colloidally stable MO NCs with controlled size, shape, host composition, and predefined dopant content have been developed over several decades to engineer specific functionalities.

### 2.1 Water Based Sol–Gel

The earliest solution routes to metal oxides were based on aqueous sol–gel chemistry, in which metal alkoxides or salts are hydrolyzed and condensed with a polymerization-like process of alkoxides (polycondensation) to form extended M-O-M (metal-oxide-metal) networks.^41^ Among these, classical sol–gel approaches, such as the Stöber method for silica-based systems,^42^ remain particularly relevant. The hydrolysis and condensation steps are strongly influenced by pH, water-to-alkoxide ratio, and the presence of chelating ligands.^43^ In contrast, late transition metal oxides (Fe, Co, Ni, Cu, Zn) generally require more careful synthetic control than early transition metal oxides because they more readily accommodate defects and mixed valence, and they can undergo competing reduction, oxidation, or hydroxide formation during reaction. As a result, their synthesis is often governed not only by precursor hydrolysis and condensation, but also by redox balance, ligand chemistry, and oxygen activity in the reaction medium. So more typical routes adopted for such oxides are, for example, precipitation-based methods^44^ or polyol processes.^45^ Moreover, classical sol–gel methods proved extremely powerful for preparing bulk gels, glasses and thin films, but they were not well-suited for producing truly colloidal, monodisperse nanocrystals: nucleation and growth were rarely decoupled, and uncontrolled aggregation or gelation frequently limited control over size and morphology.^46^ For early oxide nanocrystals, sol–gel derived particles were often polydisperse (size distribution over nm-μm, Figure 1a), poorly stabilized by surface ligands, and difficult to disperse in nonpolar solvents. These limitations motivated the development of nonaqueous and nonhydrolytic sol–gel routes, which replaced water with organic oxygen donors and exploited condensation reactions that proceed at lower water activities and higher temperatures.^47^

**Figure 1:**
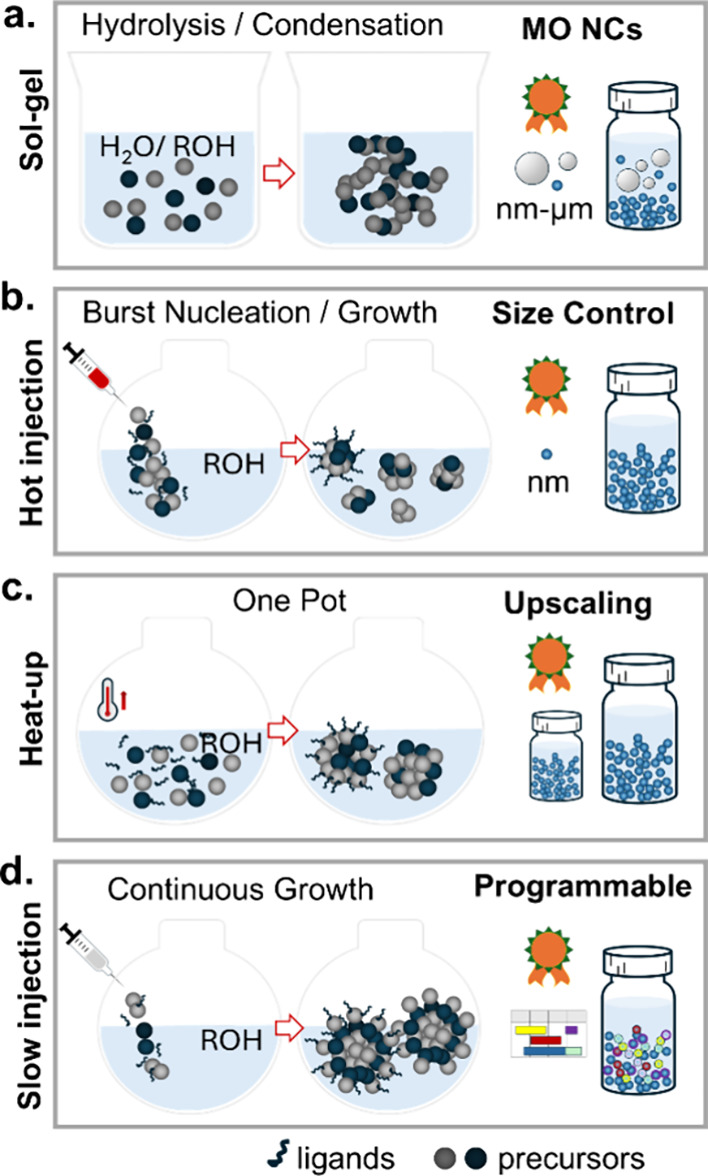
Chronological overview of metal oxide nanocrystal synthesis strategies. From top to bottom, the schematic illustrates the historical and conceptual evolution of synthetic approaches for the synthesis of metal oxide nanocrystals. Left side displays a sketch of the main synthesis mechanism while the right side depicts the relative main goal. (a) Early aqueous or alcoholic sol gel routes (H_2_O/ROH), typically defined by a hydrolysis- and condensation-based mechanism, yield limited control over nucleation and growth, often resulting in aggregation or broad size distributions. The synthesis results in metal oxides micro and nano sized particles (depicted as blue spheres), including bigger scattering centers (represented in white). (b) Subsequent adaptations to organic media (ROH) and the introduction of stabilizing ligands improve dispersion and size control on nanometric scale via hot-injection synthesis (depicted as red syringe) where rapid reactant injection promotes burst nucleation by controlled growth. (c) Heat-up variation can help in obtaining, in one pot, higher yield and scalability (up-scaling). (d) Finally, in slow injection strategies a continuous growth mechanism allows precise control over nanocrystal composition with designed dopant incorporation, offering a new perspective for functional metal oxide nanocrystals production (programmability). The main outcome is represented by a scheduled synthetic plan allowing to obtain particles with specifically designed properties.

### 2.2 Nonaqueous Routes

A major conceptual leap came with nonaqueous sol–gel chemistry, in which metal precursors react in organic solvents via mechanisms such as alcoholysis, ether elimination and esterification to generate metal-oxo networks and, under appropriate conditions, discrete nanocrystals.^48–52^ Niederberger and co-workers showed that reactions of metal chlorides, acetates or alkoxides in coordinating organic solvents (e.g., benzyl alcohol, oleyl alcohol, long-chain glycols) can yield well-crystallized oxide nanocrystals at 150–300 °C without added water, plus excellent colloidal stability after ligand exchange *(Niederberger synthesis)*.^49–52^ These nonaqueous routes brought several advantages that directly addressed the shortcomings of classical sol–gel. First, they improved size and shape control, because nucleation can be separated in time from growth by tuning precursor solubility, ligand coordination, and reaction temperature. In addition, such methods allowed better colloidal stability, as long-chain ligands are present from the outset, providing a capping layer to nascent nanocrystals and preventing uncontrolled aggregation. The importance of precursor chemistry became evident in early case studies. Narayanaswamy et al. demonstrated that In_2_O_3_ nanodots and “oriented-attached” nanoflower structures form from indium carboxylates via hydrolysis and alcoholysis, rather than direct pyrolysis of the metal carboxylates.^53^ The presence of alcohol promotes esterification and generates reactive metal-oxo species that slowly condense and crystallize. In this and related systems, the structure and reactivity of metal-carboxylate clusters dictate whether nucleation is abrupt or continuous and whether growth proceeds by monomer addition or by oriented attachment. More broadly, nonhydrolytic sol–gel approaches in organic media have been systematized for a wide range of oxides including TiO_2_, ZrO_2_, HfO_2_, SnO_2_, In_2_O_3_ and complex mixed oxides.^49–52^ These routes provided a bridge between classical sol–gel and modern colloidal nanocrystal synthesis, highlighting how ligand design and precursor speciation can be exploited to tune nucleation, growth and dopant incorporation.

### 2.3 Hot Injection

In parallel with sol–gel developments, the hot-injection method emerged from semiconductor nanocrystal synthesis, most famously for CdE (E = S, Se, Te) systems.^54^ In a canonical hot-injection experiment, a highly reactive precursor solution (often containing chalcogenide sources and metal complexes) is rapidly injected into a hot coordinating solvent. The instantaneous increase in monomer concentration is designed to exceed a critical supersaturation, giving rise to a short nucleation burst followed by a growth period at lower supersaturation, in line with the LaMer model.^43,55^ For oxides, direct transposition of hot injection is less straightforward because oxide formation often involves multistep conversion of relatively stable precursors (e.g., metal carboxylates, alkoxides) into reactive metal-oxo monomers. However, hot-injection analogues have been successfully devised, for example in ZnO syntheses where a base is rapidly injected into a zinc precursor solution in alcohol.^19^ Here, a transient regime of high supersaturation can indeed yield nucleation behavior reminiscent of LaMer, especially when the base is strong and supersaturation is achieved within milliseconds. Hot-injection has several strengths. It can deliver excellent size-focusing and narrow dispersity when nucleation is well separated from growth (Figure 1b). The rapid temperature and concentration jumps can overcome kinetic barriers to nucleation, allowing access to very small crystalline domains. It is conceptually simple and has been widely optimized for diverse semiconductor families. But it also has important limitations. Scaling up is intrinsically difficult because reproducing a sharp injection event in large volumes is challenging.^19^ The method can be sensitive to mixing and heat transfer, leading to batch-to-batch variability. For oxides, where monomer formation is not instantaneous, hot-injection often fails to deliver true burst nucleation; instead, persistent clusters and continuous nucleation are observed.^56^

### 2.4 Heat-Up

To address scalability and reproducibility, heat-up (noninjection/one pot) methods were developed (Figure 1c), in which all reagents are combined at room temperature and then heated until nucleation and growth occur.^57^ Historically, heat-up routes were thought to intrinsically yield broad size distributions because nucleation and growth overlap in time. However, detailed contemporary work, especially on ZnO and iron oxide, has shown that careful control over precursor reactivity and heating rate can deliver monodisperse nanocrystals even in heat-up mode.^19,51,58^ A striking example comes from iron oxide. NMR relaxation studies revealed that in a heating-up synthesis of Fe_3_O_4_, the presence of sufficient oleic acid can induce LaMer-like nucleation: at a critical temperature, fast esterification and precursor activation suddenly generate reactive monomer, triggering a quasi-burst nucleation even though no mechanical injection occurs.^51^ Oleic acid thus acts as a chemical “fuse” that delays nucleation until a particular temperature is reached, indicating that nucleation is governed by the onset of precursors reactivity rather than by the physical act of injection. When heat-up reactions are performed with fast heating rates, the induction period is short, and nucleation can be relatively localized in time; when heating is slow, nucleation tends to be more continuous. Successful heat-up syntheses of II–VI and oxide nanocrystals have shown that burst nucleation is not a prerequisite for monodispersity; instead, strongly size-dependent growth kinetics and controlled monomer flux can compensate for continuous nucleation.^58,59^

### 2.5 Programmable Metal Oxide

Recently, continuous-growth synthesis demonstrated a decisive advance in the historical development of MO NC fabrication, introducing a level of compositional programmability that was not achievable with earlier methods (Figure 1d).^12,13^ In this approach, dose-controlled precursor delivery enables the chemical composition of nanocrystals to be dynamically programmed during growth, allowing deterministic control over stoichiometry and dopant placement, representing a fundamental step forward in oxide nanocrystal synthesis.

Before addressing continuous growth, however, it is useful to establish a broader framework by identifying the key parameters governing morphology, composition, and dopant incorporation in MO NCs. These design parameters underpin the concept of “programmable” nanocrystals, i.e., systems whose properties can be deliberately tuned toward targeted functionalities.^60^ In the following, we provide a concise overview of these variables and the synthetic strategies used to modulate them within established colloidal approaches, enabling precise control over nanocrystal structure and composition.

First, in doped metal oxides we can distinguish a “host” material which define the crystal structure, electronic band structure, and defect chemistry, thereby determining both dopant compatibility and carrier density origin mechanisms. Representative “hosts” include indium oxide (In_2_O_3_), titania (TiO_2_), zinc oxide (ZnO), cadmium oxide (CdO), tungsten oxide (WO_3_), iron oxide (Fe_2_O_3_), stannic oxide (SnO_2_), copper oxide (CuO), manganese oxide (MnO).^17,20^ Dopant identity and incorporation mechanisms directly control carrier density. Beyond overall concentration, dopant placement, i.e., the spatial distribution and activation of dopants within the nanocrystal, is another first order critical design parameter. Dopant distribution, in fact, modifies the local dielectric environment and carrier localization, strongly influencing the optical response, as discussed in Section 4. Closely related, defect engineering represents an additional and often dominant degree of control. In many MO systems, oxygen vacancies act as intrinsic donors and strongly affect optoelectronic properties (e.g., TiO_2_,^24^ WO_3_ ^61^). Recent work has demonstrated that defect populations can be synthetically programmed. For example, during continuous growth of In_2_O_3_, Fe_2_O_3_, and CdO, the introduction of spectator salts enables controlled formation of oxygen vacancies independently of morphology.^62^ This strategy allows oxygen deficiency to be tuned directly at the precursor stage, without requiring control over oxygen partial pressure, and can induce emergent properties such as photoluminescence in otherwise nonemissive systems (e.g., black In_2_O_3_). Surface chemistry constitutes another critical parameter. The high surface-to-volume ratio of MO NCs makes their properties highly sensitive to surface states, which are central to catalytic activity and surface-mediated processes. At the same time, control over morphology and crystal anisotropy, readily achieved in systems such as Cs_*x*_WO_3_ ^63^ and ZnO,^64^ modulates surface exposure and facet-dependent reactivity. Morphology and crystalline anisotropy introduce an additional level of programmability. Shape control can be achieved via multiple synthetic strategies, including hot-injection (through precursor selection and concentration), continuous growth (through temperature, precursor reactivity, and degassing), and heat-up methods (via precursor ratios^63,65,66^ ). Importantly, these parameters are often strongly interdependent. In TiO_2_ nanocrystals, seeded growth combined with tailored precursor and cosurfactant selection enables access to distinct morphologies, which correlate with variations in oxygen vacancy concentration and are reflected in both LSPR behavior and EPR signatures.^24,67^ In indium-doped cadmium oxide (ICO), both dopant concentration and shape, controlled via annealing temperature and reagent concentration, significantly affect plasmonic properties.^22^ Moreover, dopants can actively direct growth pathways: in Mg-doped ZnO, the initial dopant concentration determines preferential crystallographic growth directions, yielding morphologies ranging from tetrapods to nanowires.^64^

Overall, the development of programmable MO NCs can be rationalized in terms of increasing levels of control. Early synthetic approaches focused on achieving reproducible average host–guest composition, where the main challenge was incorporating dopants into the lattice without phase segregation or surface trapping. In this regime, nanocrystals were effectively treated as compositionally averaged systems, and functionality was correlated primarily with nominal dopant concentration. A second level of control emerged with the recognition that dopant location is as important as dopant amount. Continuous growth approaches demonstrated that temporally controlled precursor addition during synthesis enables predictable size evolution and, critically, radial dopant profiling. This established that spatial dopant distribution can be deliberately encoded and directly influences properties such as plasmonic response. The most advanced level of programmability treats MO NCs as engineered electronic systems in which dopants, intrinsic defects, depletion layers, and interparticle interactions collectively determine functionality. In this framework, dopants do not act independently but modify carrier activation, defect equilibria, and spatial charge distribution (see **Section 4**). Expanding the comparative framework, post synthetic approaches unlock otherwise inaccessible compositions but act primarily as modification tools rather than comprehensive design strategies. For example, codoping enables simultaneous incorporation of multiple species, such as combining cationic and anionic dopants to enhance carrier density and extend LSPR tunability. For example, fluorine doping in FICO and FSCO systems enables modulation of LSPR across the 1.5–3.3 μm range.^68^ Ion exchange and replacement reactions provide alternative routes to access new compositions and architectures. Cation exchange, analogous to processes in transition metal nanocrystals,^69^ enables transformation of CuO nanocrystals into Mn_3_O_4_, Fe_2_O_3_, CoO, and NiO.^70^ Galvanic replacement reactions extend this approach, enabling formation of complex hollow structures such as Mn_3_O_4_/γ-Fe_2_O_3_ nanoboxes and γ-Fe_2_O_3_ nanocages through selective cation substitution.^71^ Postsynthetic cation exchange offers further control over optical properties. In ICO and ITO nanocrystals, controlled cation exchange induces systematic redshifts or blueshifts of the LSPR,^72^ enabling access to compositions not achievable via direct synthesis. Lastly, seed-mediated growth introduces another level of control by enabling heterostructure formation, but typically involves discrete steps and limited continuity in compositional tuning. Metallic nanocrystals (e.g., Au, Pt, Pd, Fe, Pt) can act as seeds for MO growth (e.g., ICO), yielding heterodimer systems in which optical properties arise from the interplay between dopant concentration and seed material.^73^

These approaches highlight the wide range of parameters through which the properties of MO nanocrystals can be tuned, spanning composition, defect chemistry, morphology, and postsynthetic transformations. However, in most conventional synthetic routes, the design parameters are intrinsically coupled and often adjusted indirectly, limiting precise and independent control specifically over carrier incorporation and spatial distribution. In this regard, continuous-growth strategies represent a critical step forward, as they enable direct and dynamic control over these parameters during nanocrystal formation.

## 3 Continuous Growth: Living Nanocrystals

The continuous-growth synthesis is characterized by the injection of ligands and metal precursors at a controlled rate into a hot solvent using a syringe pump, creating monomers, which condense to nucleate nanocrystals (Figure 2a,b). The reactive hydroxyl groups enable uninterrupted growth with precise control over size, shape, chemical composition, and radial dopant placement (Figure 2c). Notably, and as will be outlined further below, control over composition affects the electronic structure, vacancy chemistry and band alignment, making it a desired synthetic parameter for functional nanomaterials design (Figure 2d).^5,74,75^ The continuous-growth paradigm has emerged most clearly in nonaqueous oxide syntheses that rely on slow injection of metal oleates into hot alcohols. Hutchison and co-workers pioneered a family of reactions in which metal carboxylates are injected slowly into oleyl alcohol, where they undergo esterification to generate metal–oxo species that nucleate and grow into nanocrystals.^13,15,76,77^ The authors describe that nanocrystals grow in a quasi-“living” fashion under an approximately constant monomer flux, typically achieved by slow, continuous addition of precursor into a hot solvent. Rather than a single, short nucleation burst followed by a decaying growth stage, continuous-growth reactions are designed such that nucleation and growth unfold in a controlled and often overlapping manner, yielding monodisperse nanocrystals whose final size is determined primarily by the total amount of precursor added.^13,14^ This approach has become particularly powerful for MO NCs, though conceptually similar strategies now extend to III–V and other systems.^78,79^ Most continuous-growth syntheses of MO NCs rely on metal carboxylate (often oleate) precursors and long-chain oxygen or nitrogen donors such as oleyl alcohol or oleylamine. A prototypical example is the esterification route developed by Ito and co-workers for a broad family of oxides.^76^ In this method, metal-oleate complexes are dissolved in a suitable solvent and then injected slowly into hot oleyl alcohol (typically 200–290 °C).

**Figure 2:**
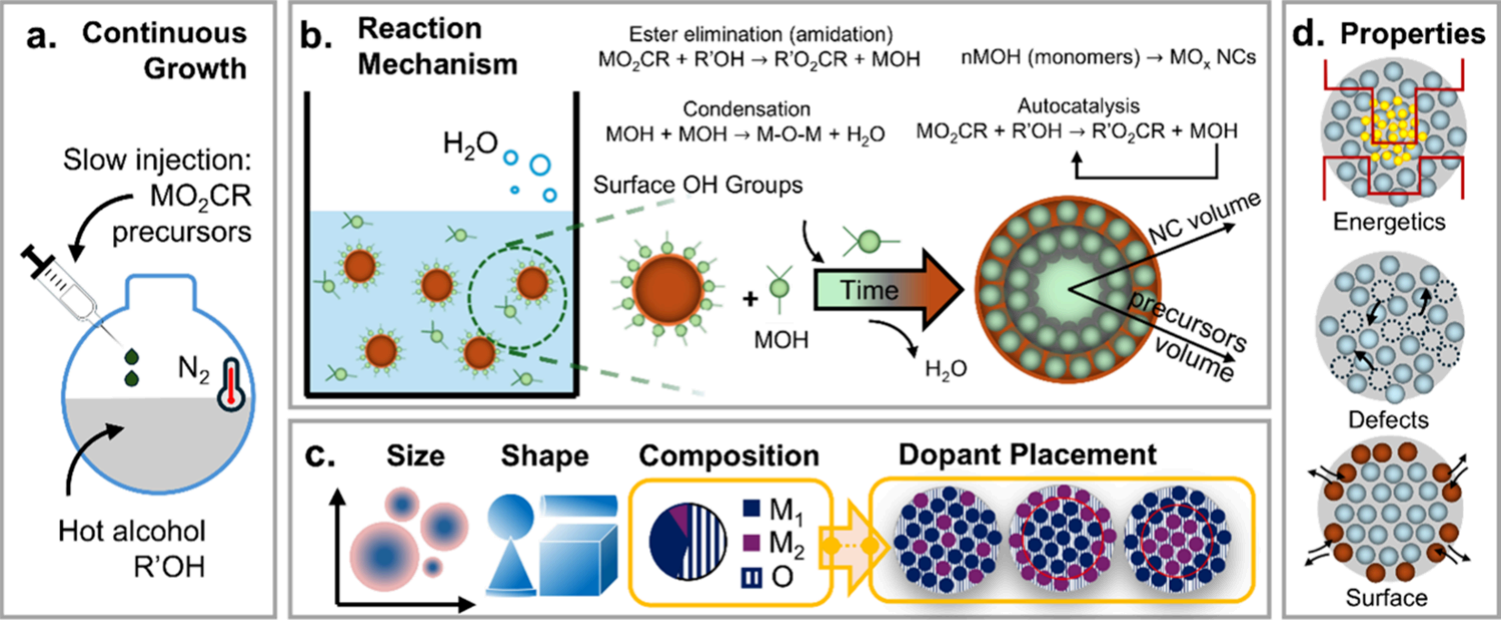
Continuous growth synthesis of metal oxide nanocrystals. (a) Schematic of a typical reaction method: ligands and metal precursors are introduced at a controlled rate into hot solvent in inert atmosphere using a syringe pump. (b) Typical chemical reactions (elimination/condensation) defining the continuous growth mechanism allowing monomers formation consumed in the autocatalytic process at the particles surface. From the left, a reaction flask during the growth process containing metal hydroxide monomers (MOH) that can interact with stable nanocrystal nuclei exposing reactive surface hydroxyl groups (−OH), generating new surface hydroxyl sites and releasing water as a byproduct, as illustrated in the center. These monomers condense to nucleate nanocrystals. On the right, representative example of nanocrystal time evolution following the continuous-growth mechanism, in which the sustained availability of reactive hydroxyl groups enables uninterrupted growth and a progressive increase in nanocrystal size while monomers are available. (c) The process enables continuous enlargement of nanocrystals, allowing precise control over the properties of size, shape and chemical composition. The layer-by-layer evolution of the nanocrystals unlocks an effective programmable dopant placement. (d) This has an impact on several electronic and optoelectronic properties such as band energy alignment, defect or vacancy formation and surface chemistry or surface states.

### 3.1 Methodology and Mechanisms

The essential chemistry of the continuous-growth mechanism of MO NCs is an esterification reaction between the carboxylate ligands and oleyl alcohol: the carboxylate is converted to an ester, and the metal is progressively transferred into metal-oxo environments that eventually condense into an oxide lattice (Figure 2).^80^ At elevated temperatures, esterification between oleate and alcohol generates hydroxo intermediates that act as reactive “monomers” for nanocrystal growth.^80^ These species condense with hydroxylated nanocrystal surfaces, propagating epitaxial, layer-by-layer enlargement. Oleate ligands play a central role in ensuring solubility of precursors and providing weak, reversible coordination to nanocrystal surfaces. This balance stabilizes colloids without blocking reactive sites.^14^ In this framework, nanocrystal surfaces are not passive sinks for monomer but instead act as catalytically active sites that accelerate precursor conversion and incorporation once crystalline nuclei are established. This surface-mediated acceleration of monomer consumption constitutes an autocatalytic growth process, consistent with a Finke-Watzky type mechanism in which slow, continuous nucleation is coupled to faster, particle-assisted growth.^58,81^ Time-resolved studies and population-balance modeling suggest that nucleation is rarely instantaneous; instead, new particles can form over extended periods while existing nanocrystals grow, with size-focusing arising from size-dependent growth kinetics rather than from an idealized separation of nucleation and growth.^82^ These clusters then condense (reaction between OH groups) and crystallize into nanocrystals without a sharply defined nucleation event. In indium oxide and doped In_2_O_3_ NCs, slow-injection esterification routes reveal continuous nucleation and growth, with reaction conditions determining whether nucleation persists or is largely confined to an early period. By combining mass-balance models with ex situ size and composition analyses, it has been shown that the steady-state monomer concentration is governed by precursor conversion kinetics and the total surface area of the growing nanocrystals.^12,14,80^

Growth is initially linear in time (constant radial growth rate), beyond which it slows dramatically because the nanocrystal surface loses reactivity, likely due to changes in cation occupancy, vacancy distribution and surface reconstruction.^77^ This behavior is consistent with a mechanism where the number of nanocrystals increases gradually early in the reaction, as clusters reach a critical size and become growth-competent. Once a quasi-steady population is established, nanocrystals grow at a rate determined by surface reaction kinetics and monomer flux, and after a certain size, changes in surface structure reduce the number of reactive sites, limiting further growth despite continued precursor addition. As a consequence the growth rate per particle decreases as the number of nanocrystals increases (for fixed precursor feed rate), and different surface ligands (oleyl alcohol vs oleylamine) or dopant precursors can either increase the number of nuclei (many small particles) or decrease it (fewer, larger particles).^83^ This mechanistic picture aligns well with a continuous-nucleation, size-dependent growth model rather than an ideal LaMer burst. Nanocrystals synthesized via continuous injection are consistently single crystalline, extending epitaxially during each growth step. This lattice continuity ensures uniformity across the nanocrystal volume during continuous injection, avoiding the defects and grain boundaries common in coalescence-driven or oriented-attachment growth. High-resolution TEM and selected-area electron diffraction confirm single-crystal integrity even in compositionally complex structures.

A key feature of these chemistries is that monomer formation is rate-limited by organic reactions at the ligand (esterification, alcoholysis, amidation). This allows the experimenter to tune monomer flux by adjusting temperature (activation of esterification/aminolysis), ligand identity and concentration (strength and lability of the metal–ligand bond), precursor structure (mononuclear vs cluster-like complexes), and injection rate (how fast precursor is delivered to the hot reaction zone). Under appropriate conditions, the monomer concentration is kept within a narrow window, avoiding large supersaturation spikes and favoring steady, size-focused growth of existing particles.^12,58^ In fact, replacing oleyl alcohol with oleylamine switches the dominant chemistry to amidation and amide elimination, which alters both the monomer formation rate and the surface chemistry of the growing nanocrystals. Comparative studies on indium oxide and ITO NCs show that switching from oleyl alcohol to oleylamine leads to different numbers of initial nuclei, different growth rates and different final sizes and shapes, even at similar temperatures and precursor feed rates.^15^ Experimental studies on metal oxide systems, including In_2_O_3_, ITO and iron oxides, demonstrate that nanocrystal size increases linearly with the total amount of precursor injected, while remaining largely independent of reaction time, provided temperature and injection rate are held constant.^77,80,83,84^ Continuous-growth strategies allow nanocrystal sizes to be tuned reproducibly across a wide range by simple adjustment of precursor dose, offering a chemically transparent route to size-control grounded in reaction kinetics rather than transient nucleation events (Figure 3a).

**Figure 3:**
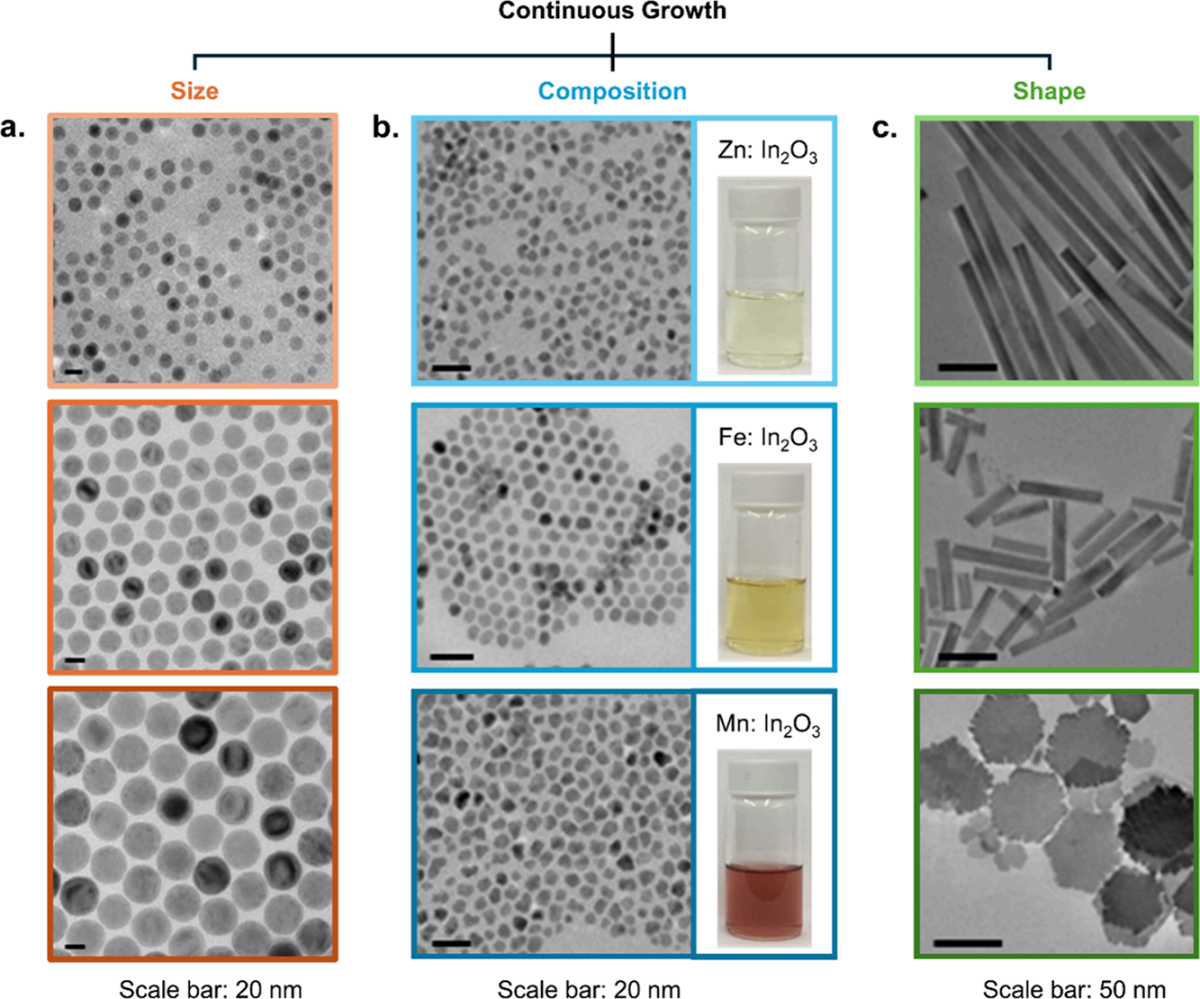
Transmission electron microscopy (TEM) images illustrating how continuous growth can be used to control (a) size, (b) composition, and (c) shape of metal oxide nanocrystals. (a) TEM images of magnetite nanoparticles synthesized via an extended LaMer approach, yielding average diameters of 12.2 ± 0.9 nm, 21.2 ± 1.1 nm, and 34.5 ± 1.6 nm (top to bottom). Adapted with permission from ref 76. Copyright 2015, American Chemical Society. (b) TEM images of doped In_2_O_3_ nanocrystals incorporating different dopant atoms, shown from top to bottom as Zn-doped, Fe-doped, and Mn-doped In_2_O_3_. The corresponding solution vials are shown on the right side of each TEM image, illustrating color changes associated with composition variation. Adapted with permission from ref 83. Copyright 2017, American Chemical Society. (c) TEM images of Cs_*x*_WO_3‑δ_ nanocrystals demonstrate the ability to obtain anisotropic particle shapes through continuous growth. Adapted with permission from ref 79. Copyright 2025, American Chemical Society.

Recent work by Gibson et al. has expanded this mechanism considerably. This study revealed that Lewis acids such as Al^3+^, Ga^3+^ and In^3+^ do not simply modify the surface chemistry of growing nanocrystals but actively catalyze monomer formation from carboxylate precursors.^85^ Mechanistically, the Lewis acid accepts electron density from oxygen ligands, thereby weakening M-O and O–C bonds within the carboxylate environment and accelerating ligand exchange and oxo-bridge formation. The consequence is a Lewis-acid-promoted metal–oxygen condensation step that dramatically increases the rate at which reactive monomers are generated. Unlike esterification alone, which is limited by organic chemistry at the ligand periphery, the Lewis-acid pathway directly enhances inorganic condensation kinetics, providing a second, independent axis for controlling monomer flux. This catalytic regulation of monomer formation has several important consequences for the mechanism of continuous growth. First, Lewis-acid catalysis alters the balance between nucleation and growth by modifying the instantaneous availability of monomers. Increasing Lewis-acid strength or loading accelerates monomer formation, raising the monomer concentration enough to generate a larger number of nuclei, leading to smaller nanocrystals at a fixed precursor dosage. Conversely, lower Lewis-acid activity or weaker catalysts suppress nucleation and yield fewer, larger nanocrystals because monomer production is slower relative to growth kinetics. Second, the Lewis-acid pathway shifts the system away from purely ligand-controlled kinetics to a regime in which metal–ligand bond polarization, precursor-catalyst complexation, and oxo-bridge formation become central kinetic determinants. These effects extend even to shape control, as demonstrated in the above-mentioned study,^85^ where varying the identity of the Group-13 catalyst modulated the morphology of Cu_2_O NCs by favoring different relative rates of nucleation, facet-selective growth, and monomer depletion.

Precursor flux emerges as an additional critical kinetic lever, controlling whether growth proceeds in a faceted, ordered manner or results in irregular, kinetically trapped morphologies.^5,13,14^ At low injection rates, in ITO synthesis, indium hydroxy monomers are supplied at low surface concentrations, allowing adsorbed species to diffuse efficiently to step edges prior to condensation. This diffusion-limited regime promotes layer-by-layer growth and yields compact, faceted nanocrystals dominated by hydroxyl-stabilized surfaces. In contrast, high injection rates generate a high surface concentration of reactive monomers, sterically hindering surface diffusion and accelerating condensation. Under these conditions, monomers nucleate new surface islands rather than extend existing steps, producing branched yet single-crystalline morphologies. These structures are kinetically trapped: strong metal–oxygen bonding suppresses shape relaxation, unlike in metallic or chalcogenide nanocrystals where postgrowth restructuring is common. Consequently, injection-controlled monomer flux, rather than postsynthetic annealing, emerges as the primary handle for morphology control.^12,14^ Recent work extended this approach involving a seed-mediated strategy. Anisotropic growth emerges once purified seeds encounter a fresh precursor environment, where precursor crowding promotes diffusion-limited deposition. As a result, nanocrystals evolve from quasi-spherical seeds into well-defined cuboidal morphologies, reflecting sustained preservation of hydroxyl-stabilized {100} surfaces under layer-by-layer growth. Under higher monomer loading, this anisotropy appears even earlier, consistent with accelerated surface deposition.^86^ Plummer et al. further showed that higher temperatures enhance surface diffusion, allowing faceted morphologies to persist under higher fluxes, whereas at lower temperatures the same flux induces extensive branching.^14^

Beyond flux, reagent identity also strongly influences facet exposure. Knecht and Hutchison compared growth in oleyl alcohol versus oleylamine, finding that amidation in amine proceeds nearly twice as fast as esterification in alcohol.^15^ Faster activation yields fewer nuclei that grow into larger nanocrystals. Surface chemistry diverges: alcohol-grown nanocrystals evolve from cubic to cuboctahedral, whereas amine-grown nanocrystals remain cubic throughout, reflecting persistent stabilization of {001} facets. XPS and annealing confirmed that oleylamine strips surface carboxylates more effectively, generating hydroxyl-rich surfaces that both accelerate precursor attachment and stabilize {001} facets. Thus, facet engineering emerges from the combined influence of flux, temperature, and reagent identity, which together dictate precursor activation and surface chemistry, and ultimately the catalytic and plasmonic properties of oxide nanocrystals.

Finally, the synthesis termination is a critical step to preserve particle size distribution, surface chemistry, and dopant homogeneity. Continuous growth routes may yield particles in dynamic equilibrium with unreacted precursors, byproducts, and excess ligands, making purification particularly delicate. Typically, the first step involves quenching the reaction to arrest further growth and dopant redistribution. Rapid cooling or dilution is essential, as elevated temperatures may promote dopant diffusion or segregation. Rapid cooling is practically implemented through immersion of the hot flask into cold-water bath or by spraying with acetone to achieve a controlled temperature drop rate.^87^ However, despite its critical role in arresting growth and fixing the final size distribution, this termination step is rarely reported with quantitative detail or systematically optimized.^12,88^ Subsequent purification generally relies on precipitation-redispersion cycles, in which an antisolvent (e.g., acetone, ethanol for particles dispersed in nonpolar solvents such as hexane, toluene) is added to flocculate the nanoparticles while leaving excess ligands and unreacted species in solution. However, for doped metal oxides, care must be taken to avoid dopant leaching, especially when dopants are weakly incorporated into the host lattice or enriched near the nanocrystal surface. Centrifugation and washing conditions must also be optimized to avoid aggregation or incomplete purification.^89^ Another key factor is the surface ligand environment. Continuous growth synthesis typically employs strongly coordinating ligands (e.g., carboxylates, amines), which remain bound after synthesis and stabilize colloidal dispersion. Postsynthetic ligand removal or exchange can alter surface chemistry and, in doped systems, may expose or redistribute dopant species^90^ (see also Section 4.2). Finally, drying and storage conditions must be carefully controlled. Exposure to air or moisture can in fact induce oxidation state changes, dopant redistribution, or surface reconstruction, particularly in late transition metal oxides.^11^

### 3.2 Versatility (Materials, Scale-Up)

The continuous-growth esterification/amidation platform has been used to synthesize a wide variety of binary and doped MO NCs, often with gram-scale yields and excellent size control. A landmark work by Ito et al.^80^ reported a lower-temperature esterification process (<230 °C) for monodisperse nanocrystals of: In_2_O_3_, ITO, γ-Fe_2_O_3_, Mn_3_O_4_, CoO, and ZnO, as well as epitaxial core/shell structures such as γ-Fe_2_O_3_/MnO and ZnO/β-Ga_2_O_3_ (Figure 3b). In all cases, a rapid esterification of metal oleates in hot alcohol produced crystalline nanocrystals in high yield, with size control obtained by varying reaction time and precursor feed. Subsequent work from the same and related groups expanded the library of oxides accessible via continuous growth. For instance, spinel iron oxide nanocrystals with tunable sizes from ca. 3–10 nm were successfully synthesized, with the growth curve revealing linear growth followed by surface-reactivity-limited saturation.^84^ Similarly, transition-metal-doped In_2_O_3_ NCs (e.g., Co:In_2_O_3_, Mn:In_2_O_3_) were obtained, incorporating the dopants substitutionally through the same esterification-driven growth process.^83^ Zr-, Ti- and Ce-doped In_2_O_3_ NCs were prepared with the same slow-injection approach at fixed nominal dopant levels while varying their sizes: this methodology enabled systematic studies on the dopant activation, surface depletion and the role of dopant electronegativity.^91^ Continuous growth also enables size control across different host material chemistries, as demonstrated for In-doped CdO^92^ and ZnO doped with substitutional trivalent cations (Al^3+^, Ga^3+^, In^3+^).^28^ Further, in γ-Ga_2_O_3_ NCs, synthesized via continuous-growth from gallium oleate in oleyl alcohol, the inclusion of sodium oleate as a spectator salt allows controlled formation of bulk vs surface oxygen vacancies.^93^ In all these systems, continuous-growth yields narrow size distributions, high crystalline quality and excellent colloidal stability, while providing a level of mechanistic transparency and synthetic flexibility that is hard to achieve in conventional hot-injection or heat-up reactions. Continuous growth methods have also been tailored to anisotropic oxides. For example, Nb_2_O_5‑x_ and Nb_12_O_29_ nanoplatelets can be selectively grown by preparing specific niobium oxo clusters as precursors and then allowing them to undergo controlled condensation and growth in coordinating solvents under conditions that favor two-dimensional anisotropy.^94^ In a different oxide family, Cs_*x*_WO_3‑δ_ NCs can be grown anisotropically by first synthesizing rod- or platelet-shaped seeds via hot injection, then continuously injecting cesium and tungsten oleate precursors at constant rate. During this continuous-growth stage, rods lengthen along the *c*-axis while platelets expand laterally, preserving anisotropy and allowing independent control of aspect ratio and overall size (Figure 3c).^79^

Although most detailed mechanistic work on continuous growth has focused on metal oxides, conceptually similar continuous-injection strategies have been applied to III–V semiconductors. Seedless continuous injection of In and P precursors into hot coordinating solvents has been shown to yield InP quantum dots with large sizes and low size dispersity, in contrast to the traditional hot-injection protocols where nucleation and growth are more difficult to decouple.^78^ In comparison to hot-injection and heat-up, the continuous-growth method thus offers a set of advantages that are particularly attractive for doped MO NCs and complex heterostructures. First, from a go-to-market perspective, continuous growth syntheses are highly reproducible and inherently scalable since the reaction temperature is kept constant, and the precursor feed is controlled. As long as precursor and solvent are supplied, growth can continue for arbitrarily long times while maintaining constant dispersity, since the number of nuclei is fixed after the initial nucleation burst. Nanocrystals of varied diameters can thus be produced with narrow size distributions, directly reflecting the living polymerization analogy of continuous mass increase without new particle formation.^12,13^ Recent work from Rebecchi et al. demonstrates that this strategy is viable beyond the laboratory, with gram-scale production of ITO NCs while retaining structural and optical quality.^95^ Similarly, Wainer et al. demonstrated the scalability delivered by continuous growth for aliovalently doped ZnO NCs.^28^ Such scalability arises because continuous injection fixes the number of growth-competent nuclei early and permits indefinitely extended layer-by-layer growth while maintaining narrow dispersity. Collectively, these features highlight continuous growth as a robust and scalable route to oxide nanomaterials, enabled by the precise precursor delivery characteristic of continuous injection and well suited for optoelectronic materials.

### 3.3 Metal Oxide Nanocrystal Doping Regulation

A central objective in nanocrystal synthesis is the deliberate control of chemical composition, which at the nanoscale extends beyond bulk stoichiometries to encompass the distribution of atoms among surface sites, subsurface regions, cores, interfaces and defect environments (see Figure 4).^96^ Composition dictates electronic structure, vacancy chemistry, band alignment and catalytic behavior, making it one of the most critical synthetic parameters for functional nanomaterials.^5,74,75^ Achieving a desired composition is nontrivial because precursor reactivity, ligand-binding equilibria, nucleation barriers and growth kinetics often bias the system away from nominal feed ratios.

**Figure 4:**
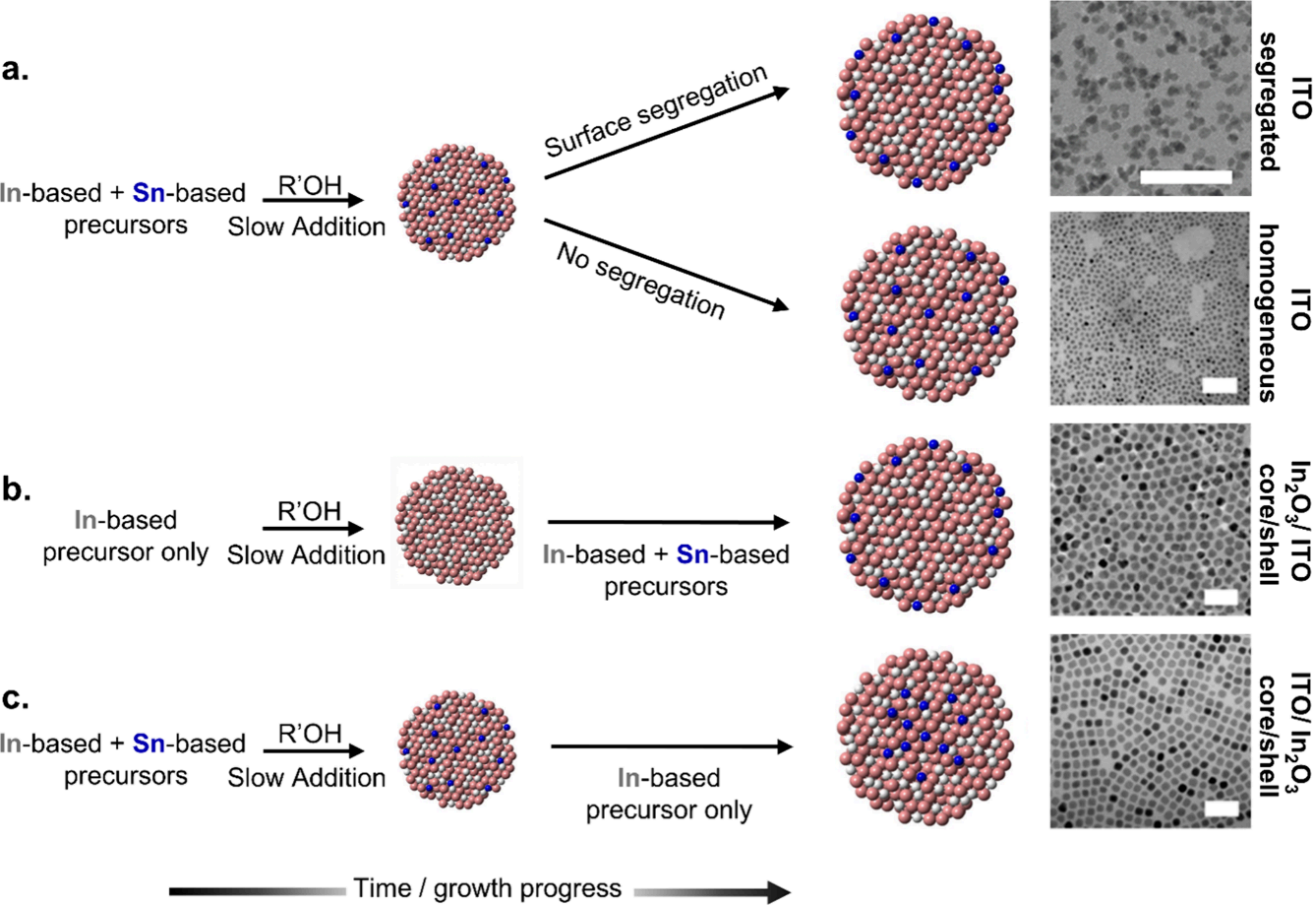
Kinetic evolution of Sn dopant distribution and extended composition in colloidal In_2_O_3_ nanocrystals. (a) Schematic growth pathways illustrating two distinct structural outcomes obtained when both In- and Sn-based precursors are present from the onset of the growth. Upper schematic: Sn is preferentially localized toward the nanocrystal surface, resulting in surface-segregated Sn distributions. Lower schematic: Sn is distributed throughout the nanocrystal, yielding homogeneously doped ITO nanocrystals. (b) Schematic growth pathway leading to core/shell In_2_O_3_/ITO nanocrystals, obtained when In-based precursor is supplied during the initial growth stage and Sn-based precursor is introduced at later stages. (c) Schematic growth pathway leading to inverted core/shell ITO/In_2_O_3_ nanocrystals. In this configuration, In- and Sn-based precursors are introduced together during the initial growth stage, forming an ITO core, followed by a second growth stage in which only the In-based precursor is supplied, resulting in the formation of an In_2_O_3_ shell. Indium, oxygen, and tin atoms are depicted as red, white, and blue spheres, respectively. Representative transmission electron microscopy (TEM) images aligned with each schematic illustrate the corresponding nanocrystal samples. Scale bars: 50 nm. TEM image of “ITO segregated” adapted with permission from ref 6. Copyright 2014, American Chemical Society. Nanocrystal schemes and TEM images for “ITO homogeneous”, “In_2_O_3_/ITO core/shell”, and ITO/In_2_O_3_ core/shell are adapted with permission from ref 96. Copyright 2017, American Chemical Society.

Metal oxides exemplify this challenge: their oxygen content, hydroxylation, and vacancy concentration vary continuously during synthesis and strongly influence optoelectronic properties such as conductivity and plasmonic response.^97–99^ As a result, chemical composition must be regarded not merely as a static input, but as an emergent property of the reaction pathway, reflecting the coupled dynamics of precursor chemistry, ligand exchange, monomer formation, nucleation and growth.^15,100^ These considerations become even more important in doped nanocrystals, where achieving both the correct overall dopant concentration and the correct spatial dopant distribution is essential. Because typical colloidal synthesis temperatures (<400 °C) do not permit bulk-like diffusion, dopant incorporation is primarily kinetically controlled.^91,99,101^ The outcome depends on the relative activation kinetics of dopant and host precursors, their coordination environment and ligand hardness/softness, and the energetics of substitution into the growing lattice.^6,102,103^ Dopant precursors that activate too slowly remain adsorbed as surface complexes or form neutral clusters without integrating into the lattice, whereas precursors that activate too rapidly may nucleate undesired secondary phases.^104,105^ Even when dopants enter the lattice, their radial distribution depends on their availability during the growth process. Indeed, early availability yields core-rich doping; later availability produces shell-biased doping; an intermediate availability modulation produces graded or multilayered dopant architectures (Figure 4a).^12,79,96^ As a result, composition control and dopant-placement control are inseparable synthetic goals. Early theoretical work proposed self-purification, suggesting that dopants and defects have higher formation energies in nanocrystals than in bulk, so that they are expelled toward the surface during growth.^102^ While attractive conceptually, this view assumes near-equilibrium conditions and substantial diffusion. In colloidal synthesis, however, temperatures are typically <400 °C, ligands stabilize surfaces, and ionic diffusion is slow, especially in oxides. Experiments across many systems now show that kinetic, not thermodynamic, factors dominate dopant incorporation.^99,103^ Buonsanti et al. systematically demonstrated that dopants are effectively incorporated only when dopant and host precursors exhibit comparable reactivity under the chosen ligand environment.^99^ If the dopant precursor reacts much slower (for instance, a hard Lewis acid such as Al^3+^ paired with hard carboxylate ligands in a softer Zn^2+^ matrix), dopants remain on the surface or form neutral complexes and do not contribute charge carriers. Conversely, when host and dopant are chemically similar (such as Sn and In in ITO), precursors can be matched so that the nominal dopant fraction in solution is faithfully transferred into the solid, yielding high dopant activation and reproducible properties.^13,106^ Stavrinadis et al. reached similar conclusions for trivalent dopants (In^3+^, Sb^3+^) in PbS quantum dots: differences in kinetic incorporation efficiency, site occupancy, and formation of charge-neutral complexes dominate over equilibrium solubility limits.^103^ Metal oxides introduce an additional constraint: cation diffusion is extremely slow at typical colloidal synthesis temperatures. For Al^3+^ in ZnO, diffusion coefficients at 900 °C are on the order of 10^–14^ cm^2^/s, implying that at 250–300 °C and over several hours, the diffusion length is well below 1 nm.^107^ This means that dopants introduced during growth become essentially trapped in the lattice, and their final positions reflect the temporal sequence of nucleation and growth rather than thermodynamic preferences. This kinetic trapping complicates attempts to equilibrate dopants by postsynthetic annealing without causing coarsening or phase segregation. For example, Ni^2+^-doped TiO_2_ prepared via sol–gel exhibits markedly different dopant distributions after aging and annealing: Ni­(OH)_2_ formed during aging segregates into NiO clusters upon heat treatment, whereas rapid annealing can “freeze” Ni^2+^ uniformly in the TiO_2_ lattice, leading to different photocatalytic behaviors.^108^ For aliovalent dopants in MO NCs (e.g., Al^3+^ in ZnO, Sn^4+^ in In_2_O_3_), this means that dopant activation and spatial distribution must be engineered during the original synthesis, by controlling precursor chemistry, solvent type, ligand environment, growth pathway, and any etching/regrowth steps. Zhou et al., for example, developed a single-pot protocol for Al-doped ZnO (AZO) in which initial AZO NCs are grown at high temperature, then partially etched by oleic acid at a slightly lower temperature.^105^ Etching reduces core size and rereleases Zn and Al oleates into solution with a higher Al/Zn ratio compared to the earlier stage. Subsequent regrowth incorporates Al more efficiently, leading to higher fractions of active dopants. More recent work extended these concepts to a family of In_2_O_3_ NCs doped with Sn, Zr, Ti, Ce and other cations at fixed nominal dopant concentrations but varying sizes.^91^ Here, careful analysis of dopant activation and electron densities highlighted the role of dopant electropositivity and accessible oxidation states. For example, Zr dopants, despite being somewhat surface-segregated, exhibited the highest dopant activation due to favorable donor level alignment, whereas Ti dopants, even when core-segregated, showed poor activation due to their propensity for mixed valence (Ti^3+^/Ti^4+^). The same continuous growth platform has been used to engineer intrinsic defect distributions. In γ-Ga_2_O_3_ NCs, adding sodium oleate promotes formation of bulk oxygen vacancies rather than surface vacancies, as evidenced by XPS and photoluminescence analysis.^93^ Bulk vacancies behave as deep donors that localize electrons and enhance UV emission, while surface vacancies are doubly ionized and results in donor–acceptor pair (DAP) emission; thus, changing defect location and charge state strongly modulates photophysical behavior. This is conceptually analogous to doping, but with intrinsic defects acting as the “dopant species”.^98^

Continuous slow-injection esterification synthesis provides a particularly powerful and much less intricate platform for deliberate radial dopant placement in oxide nanocrystals (Figure 4). Because growth proceeds approximately layer-by-layer and is proportional to the amount of precursor added, temporally changing the dopant composition of the injection solution maps directly onto radial composition profiles. Hutchison and co-workers demonstrated this explicitly for ITO NCs.^96,106^ Different core/shell nanocrystal systems based on ITO and In_2_O_3_ have been produced via continuous layer-by-layer enlargement.^96,97^ By using sequences of undoped and doped precursor injections, they synthesized: core-doped NCs (Sn-rich cores with undoped shells), uniformly doped NCs, and shell-doped NCs (undoped cores with Sn-rich shells), all with similar overall sizes and narrow dispersity (Figure 4b,c). These different radial architectures have profound impact on their optical and electronic properties and hence can be used to program their optoelectronic response to desired characteristics (more details reported in Section 4). The deterministic placement of dopants allows creating complex architectures ranging from core/shell structures to graded NC architectures, but enables also the control over dopant activation.^86,96,107,109^ These results underscore that dopant placement, shaped by the interplay of precursor reactivity, flux, and diffusion, acts as an independent design parameter in MO NCs rather than a passive consequence of overall composition and that the continuous-growth method provides a valuable tool to control it.

## 4 Programmability Control Through Doping

In parallel with advances in synthesis protocols, significant efforts have focused on engineering doped MO NCs with specifically tailored properties and functions. As stated in Section 2.5, programmability arises from the interplay between structural parameters (size, shape, crystal structure), dopant characteristics (presence, type, incorporation mechanism, distribution, and activation), and surface-related chemistry (ligands, defects, solvent environment). Together, these factors determine the resulting optical, electronic, and catalytic properties that make these materials so unique. Among these properties, dopant placement has emerged as a particularly powerful strategy for tuning optoelectronic behavior while preserving the host lattice. For this reason, many recent studies focus on the control of dopant distribution.^12,19,20,106,110^ Dopant placement is inherently a multiscale problem that includes several key questions: whether dopants are present, how they are incorporated into the host lattice (e.g., substitutional vs interstitial vs surface-bound), their local coordination and oxidation state, and their spatial distribution (uniform, clustered, interfacial, or radially graded). This information is hardly provided by a single-technique approach. In addition, establishing correlations between structure, dopant configuration, surface chemistry (largely determined by synthesis) and the resulting properties requires a comprehensive, correlative characterization approach. Therefore, mapping the structural-property landscape of doped MO NCs typically requires a combination of complementary techniques, including electron microscopy, ensemble composition/size analysis, crystallographic techniques, surface- and ligand-related probe techniques, spectroscopy methods and electrical characterization.^111^

### 4.1 Metal Oxide Nanocrystal Characterization

Imaging techniques such as transmission electron microscopy (TEM) and high-resolution TEM (HR-TEM) are routinely used as first-level characterization tools to determine nanocrystal size, shape, and lattice structure at the single-particle level, while SEM is typically employed for NC films.^112,113^ To improve statistical relevance and probe larger sample volumes, small-angle X-ray scattering (SAXS) is widely used, particularly for monitoring nucleation and growth processes in situ.^114^ Accurate compositional quantification is typically achieved by inductively coupled plasma optical emission spectroscopy or mass spectrometry (ICP-OES/MS),^115^ which provide the average dopant content after purification and are essential for distinguishing true incorporation from precursor excess. However, these methods do not provide information on dopant location or incorporation mechanism. Crystallographic characterization is commonly performed using selected-area electron diffraction (SAED)^116^ and X-ray diffraction (XRD).^117^ SAED provides local information on phase and crystallinity but suffers from limited statistical representativeness, while XRD offers ensemble-averaged structural information, enabling phase identification, assessment of phase purity, and detection of lattice distortions. In NCs, peak broadening, small size, and low dopant concentrations significantly limit sensitivity to dopant incorporation, making XRD primarily a screening tool rather than a definitive probe of dopant distribution. To directly probe dopant incorporation and local environment, more sensitive, element-specific techniques are required. Electron microscopy coupled with Energy-dispersive X-ray spectroscopy (TEM-EDX/STEM-EDX)^118^ enables spatially resolved compositional mapping and is widely used to identify radial gradients, interfacial segregation, or core–shell structures. While highly effective for heavier dopants, its sensitivity decreases at low dopant concentrations or when atomic numbers are similar, as in the case of Sn and In in ITO NCs. Electron Energy-Loss spectroscopy (EELS) can further enhance chemical sensitivity and, in favorable cases, provide insight into oxidation state and bonding.^119^ X-ray-based techniques offer complementary, element-specific structural information. X-ray photoelectron spectroscopy (XPS), including angle-resolved modes, provides surface-sensitive compositional and chemical-state information, enabling identification of oxidation states and surface chemistry.^6,120,121^ X-ray absorption techniques such as XANES and EXAFS^122^ are particularly powerful, as they directly probe the local coordination environment, oxidation state, and bond distances around dopant atoms, helping to distinguish substitutional incorporation, interstitial defects, and segregated phases. Nevertheless, these X-ray-based techniques are often ensemble-averaged or limited in lateral spatial resolution by X-ray diffraction limits, i.e., typically tens to hundreds of micrometers, making again dopant mapping challenging. Higher spatial resolution, especially with hard X-rays, generally requires synchrotron-based microscopy.^123^ Recent work has further shown that energy dependent XPS (ED-XPS) can directly reveal near-surface band bending in plasmonically active MO NCs, highlighting surface electronic gradients that can strongly influence LSPR behavior.^124^ Electron paramagnetic resonance (EPR) can be used to elucidate the paramagnetic nature of doping or defects centers, being highly sensitive to oxidation state and local symmetry, as well as to defect states such as oxygen vacancies.^125,126^ For example, in ZnO NCs not all dopants introduce the same doping centers, but isovalent doping with (Co^2+^, Mn^2+^, Ni^2+^) can introduce paramagnetic centers.^19^ Analogously, the oxygen vacancies which give paramagnetic behavior in ZnO have been correlated to the appearance of green photoluminescence through combination of EPR and photoluminescence/absorbance measurements.^23^ Nuclear magnetic resonance (NMR) is primarily used to probe surface chemistry, providing detailed insight into ligand binding, exchange dynamics, and surface reactivity.^127–130^ Colloidal properties and surface interactions can be assessed by dynamic light scattering (DLS),^131^ which measures hydrodynamic size and aggregation behavior, while thermogravimetric analysis (TGA)^132^ is used to quantify ligand coverage and stability. Zeta potential measurements provide information on surface charge and colloidal stability at the macroscale. Scanning probe techniques, such as scanning tunnelling microscopy (STM), electric force probe microscopy (EFM) and Kelvin probe force microscopy (KPFM), are employed for assessing the surface charges and potential in single NCs or assembled films.^105,133^ Even considering this extensive range of methods, structural characterization alone is often insufficient to fully validate dopant placement in functional nanocrystals, as many of the most critical effects are electronic rather than purely structural. Optical and spectroscopic techniques provide a complementary and often more sensitive probe of functional behavior.^20^ Fourier-transform infrared (FTIR) spectroscopy provides complementary information on ligand vibrational modes in the mid-infrared and can also probe plasmonic responses in the NIR–SWIR range. As indirect techniques, these methods are typically combined with structural approaches such as XPS, STEM-EDS, or STEM-EELS to validate the observed optoelectronic features. In addition, magnetic circular dichroism (MCD) spectroscopy can be employed to probe magneto-optical responses, and disentangle free-carrier, interband, and dopant-related contributions to the optical response. Because MCD measures the differential absorption of left- and right-circularly polarized light under an applied magnetic field, it is particularly sensitive to magnetically split electronic states and carrier spin polarization and can therefore provide insight into dopant or defect-induced electronic states.^119,134,135^ Finally, for emissive systems, photoluminescence (PL) spectroscopy offers insight into recombination pathways and defect states, often associated with dopant-induced levels or intrinsic defects such as oxygen vacancies.^136–138^ To achieve higher spatial resolution, cathodoluminescence (CL) can also be employed, where nanocrystals are excited by an electron beam in a scanning electron microscope. This approach enables mapping of radiative recombination processes with nanometer-scale precision, allowing emission features to be directly correlated with local defects and dopant distributions.^139^

The broad characterization toolbox above-mentioned can provide information on morphology, composition, coordination and dopant spatial arrangement, but not sufficient for tackling the multiscale relationship between dopant placement, dopant activation and the resulting ensemble-level optoelectronic response, especially in plasmonic NCs. Accessing how these features collectively define the electronic structure, in view of programmable functionalities for specific applications, also requires probes which are sensitive to the ensemble nanocrystal response. Therefore, combination of elemental and microscopy-based techniques with optical spectroscopy (UV–vis-NIR absorption^20,140^ ) and modeling is instrumental in correlating doping with particles properties. In doped MO NCs, the LSPR is inherently sensitive to variations in free carrier density, carrier damping, and the local dielectric environment, all of which are intimately linked to dopant activation and spatial distribution. For this reason, the following discussion focuses on interpreting dopant placement and distribution through LSPR modifications, in combination with theoretical modeling, as a central approach to accessing the dopant-dependent electronic structure in plasmonically active MO NCs.

### 4.2 Dopant Activation and Placement in Metal Oxide Nanocrystals—Effect on the Optical Response

In doped MO NCs, the high density of free carriers gives rise to a localized surface plasmon resonance, i.e. the collective oscillation of conduction-band electrons driven by an external electromagnetic field and strongly confined by the nanocrystal geometry and dielectric environment. In particular, the LSPR signatures, readily obtained from UV–vis absorbance, enable quantitative correlations between the observed spectral features and the underlying physical parameters and their dielectric function (typically described by the Drude response). Importantly, these parameters can vary locally within the volume of the NC. In fact, in contrast to noble metals, doped MO NCs display a richer plasmonic response, where even modest variations in synthesis conditions can strongly affect the optical and electronic properties, making rigorous control over synthesis parameters very important.

In conventional noble metal nanoparticles (Au, Ag, Pt, Pd), the free-electron density is set by the bulk conduction electron density, as described by the Drude model, and can only be modestly modified by changing the elemental composition or alloying.^141^ As a result, for a given size, shape, and dielectric environment, both the LSPR energy and line width are determined. Surface modification of noble metal nanoparticles offers only limited control over the free-electron population and mainly tunes the local dielectric environment and damping.^142^ The high carrier density in noble metals (10^22^ ÷ 10^23^ cm^–3^) also implies subnanometric electrostatic screening. Therefore, upon photoexcitation the conduction electrons can be treated, to first approximation, as spatially quasi-uniform electron gas over nanometric distances, which limits the possibility to engineer nanoscale spatial variations in carrier-density gradients.^143^ Doped metal oxides, by contrast, operate at lower carrier densities within a wide-band gap semiconducting host. This makes the dopant-induced free-carrier concentration an adjustable design parameter, enabling flexible modulation of the plasmonic response. At constant size, increasing dopant density and the associated free-carrier concentration increases the plasma frequency, which shifts the zero crossing of the real part of the Drude dielectric function to higher energies and, in turn, tunes the LSPR.^7,144^ In addition, the typically wide band gap of MO NCs pushes interband transitions to higher energies, well above the plasmonic resonance, resulting in comparatively lower losses and narrower LSPR features.^145^ In this context, dopant activation is essential because it determines the effective carrier density that actually contributes to the plasmonic response.^146^ Eventually, in doped MO NCs the carrier density is not necessarily uniform or fixed. Beyond dopant concentration, oxide surfaces support rich ligand and adsorbate chemistries that can be exploited to modulate carrier density via e.g. electrochemical, photochemical or chemical charging.^8,20,147^ Moreover, carrier density can spatially vary at the nanoscale due to dopant placement and surface band-bending effects induced by Fermi-level pinning at the nanocrystal surface.^97,148^ All these parameters are controlled by synthesis, including continuous-growth synthesis, delivering an additional tuning knob to control their optical and electronic properties.

In doped semiconductors, and especially in doped MO NCs, the nominal doping level is set by the fraction of dopant atoms incorporated into the host lattice during synthesis. For example, in ITO NCs, the LSPR originates from a high density of delocalized conduction-band electrons generated primarily by aliovalent substitution of Sn^4+^ on In^3+^ lattice sites. Each substitute Sn donor nominally contributes with one extra electron, thus providing an upper bound to the free carrier density. Once the electron density reaches sufficiently high values (typically 10^20^ ÷ 10^21^ cm^–3^), ITO NCs behave as degenerate semiconductors and exhibit Drude-like intraband dielectric response in the near-infrared.^149,147,20^ When this response is confined to a nanoscale volume, it gives rise to a plasmonic resonance and a LSPR peak in the visible to near-infrared spectral range. Simultaneously, increasing dopant and defect densities enhances carrier scattering, which broadens the LSPR and therefore demands precise synthetic control.^8^ Importantly, the carrier density that effectively contributes to the plasmon depends on dopant activation. The fraction of Sn dopants that donates mobile electrons is sensitive to their local coordination and radial distribution within the NC, and it can be reduced by compensation and trapping associated with lattice defects, such as oxygen vacancies or cation vacancies.^6^ As a result, the plasmonic response is governed by the effective electron concentration rather than the nominal aliovalent doping level.

In practice, both dopant activation and dopant placement are dictated by the details of the synthesis, including the choice and relative reactivity of the precursors, as well as the thermal profile of the reaction. As reported in early studies,^21,150^ heat-up syntheses of ITO NCs can yield markedly different optical response despite similar nominal doping levels. Changes in LSPR shape and energy were attributed to differences in dopant activation and in the radial dopant distribution.^8,21,150^ Consistent with this picture, dopant activation in ITO NCs can be triggered by overgrowing a dopant-free In_2_O_3_ layer, which increases dopant activation and markedly reduces LSPR damping. In these core/shell structures, the peak line width decreases compared to nonshelled nanocrystals and the Drude damping can reach values as low as ≈ 1600 cm^–1^ (≈ 0.2 eV), consistent with reduced impurity scattering within the dopant-depleted shell.^96^

To mitigate the strong and often poorly controlled coupling between dopant segregation, activation, and scattering that characterizes heat-up syntheses, continuous-growth methods provide an effective alternative. Unlike heat-up routes, these protocols make it possible to keep NC size and total dopant content approximately fixed while deliberately designing the radial dopant architecture. As discussed in Section 3 3, Hutchison and co-workers prepared a series of particles with similar size and overall composition, spanning homogeneously doped nanocrystals, ITO cores with undoped In_2_O_3_ shells, and In_2_O_3_ core with ITO shells (In_2_O_3_/ITO).^96^ Additionally, shell thickness was varied by extending the growth time or adjusting the amount of precursor introduced during the second growth stage, enabling structural control that is essentially inaccessible in simple heat-up syntheses. Building on earlier observations by Lounis et al.,^6^ Gibbs and co-workers^109^ demonstrated, with improved control over the synthetic protocol, that dopant placement alone can yield qualitatively different plasmonic responses at fixed nanocrystal size and overall composition (Figure 5a–c). Core-doped nanocrystals exhibit either a single LSPR resonance or a dual-mode response depending on the thickness of the undoped shell (blue dotted and dashed lines in Figure 5a,c). Shell-doped architectures, where free carriers are concentrated in the shell, display two well-resolved LSPR modes whose energies and relative intensities evolve with shell thickness (red dotted and dashed lines in Figure 5a,c).^109^ These results show that, in continuous-growth syntheses, radial dopant segregation by itself can generate and tune multiple plasmon modes, establishing dopant architecture as an independent design parameter for multimode plasmonic resonances beyond what is accessible in uniformly doped MO NCs. More broadly, comparisons between heat-up and continuous-growth syntheses highlight that the optical response of doped MO NCs cannot be interpreted independently of the processing and growth conditions. Indeed, these conditions set dopant activation, dopant placement (radial distribution), and the associated scattering processes, which in turn govern the LSPR energy, line width, and line shape. Precise control of synthesis is therefore needed for robust interpretation and cross-comparison of plasmonic properties.

**Figure 5:**
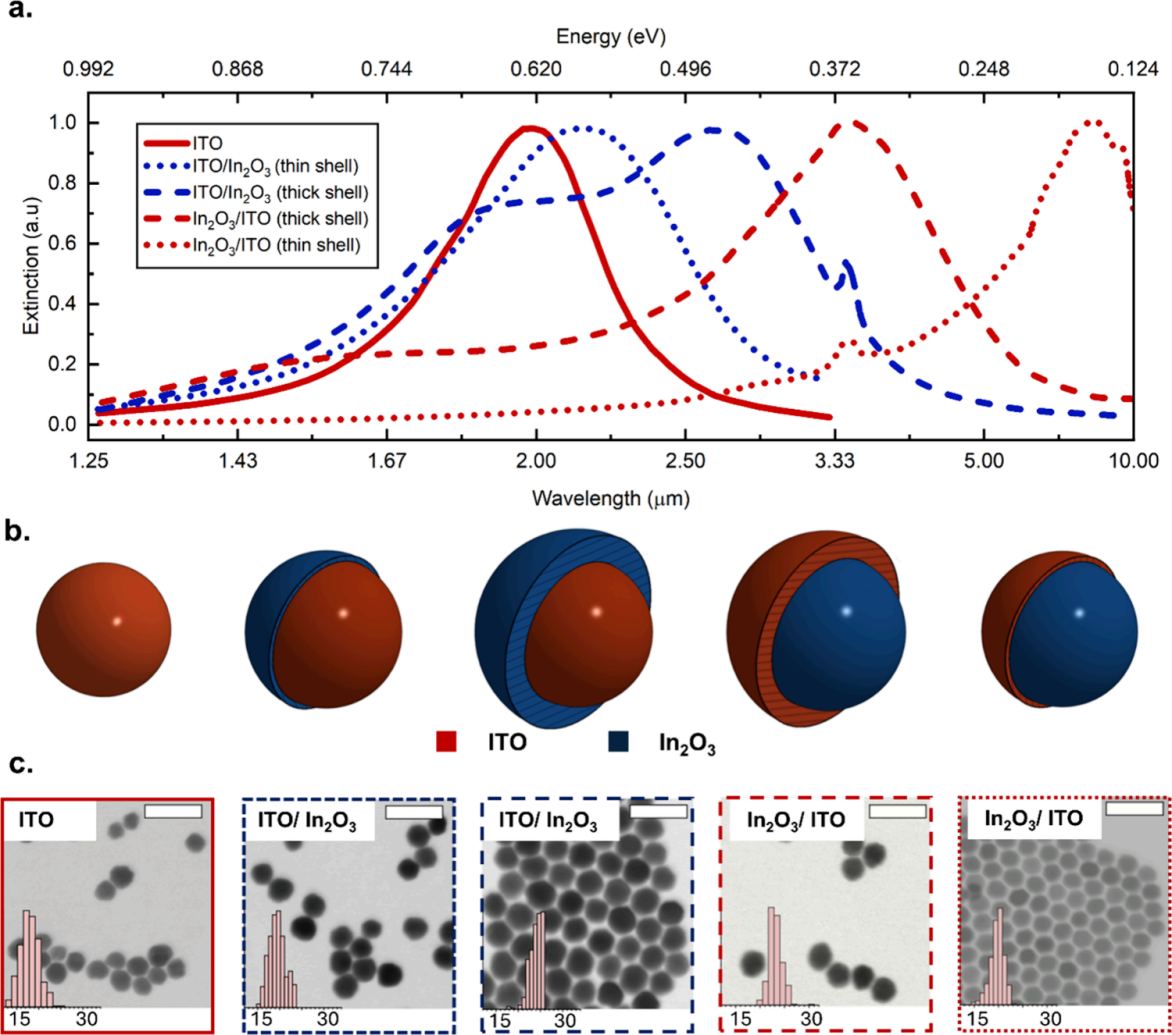
Optical properties, structural schematics, and electron microscopy characterization of Sn doped In_2_O_3_(ITO) nanocrystals and core/shell nanocrystals with both ITO/In_2_O_3_and In_2_O_3_/ITO architectures. (a) Normalized experimental extinction spectra of homogeneously doped ITO nanocrystals (radius ≈ 9 nm, solid red line), core-doped ITO/In_2_O_3_ nanocrystals with different In_2_O_3_ shell thicknesses (0.7 nm, blue dotted line; 3.4 nm, blue dashed line), and shell-doped In_2_O_3_/ITO nanocrystals with different ITO shell thicknesses (0.7 nm, red dotted line; 2.6 nm, red dashed line). (b) Schematic representations of the corresponding nanocrystal architectures, illustrating homogeneous doping and core/shell configurations; red and blue regions denote ITO and In_2_O_3_, respectively. (c) STEM micrographs and corresponding size distributions of ITO and core/shell nanocrystals. The color and line style used to frame each image match those used for the extinction spectra in panel (a), and the micrographs are vertically aligned with the schematics in panel (b), enabling direct visual correlation between optical response, nanocrystal architecture, and morphology. Scale bars in STEM micrographs are 50 nm. Adapted with permission from ref 109. Copyright 2020, American Chemical Society.

### 4.3 Post-Synthesis LSPR Tunability

A key advantage of doped MO NCs is that their plasmonic response can be actively tuned after synthesis. Because their carrier densities typically lie in the 10^20^ ÷ 10^21^ cm^–3^ range, the LSPR is highly sensitive to even small changes in free-carrier concentration.^20,147^ Accordingly, beyond dopant control during growth, doped MO NCs enable postsynthetic modulation of carrier density through electrochemical, photochemical or chemical redox processes, which directly translates into tunable optical properties (Figure 6). Electrochemical charging and discharging, implemented by applying an electrochemical potential to MO NC solutions or films, provides a dynamic plasmon tuning, as displayed in Figure 6a–c. In particular, Figure 6a shows the *in situ* spectro-electrochemical cell used to record time-dependent extinction spectra of nanocrystals under applied bias. Reduction increases the LSPR absorption and is accompanied by a blue shift (Figure 6b). On the other hand, oxidation reverses this trend, attenuating the LSPR intensity and inducing a red shift (Figure 6c). A complementary postsynthetic strategy is photodoping, where continuous above-bandgap illumination generates electron–hole pairs and, in the presence of a sacrificial hole scavenger (e.g., EtOH), holes are removed to suppress recombination and enable net accumulation of long-lived delocalized electrons and thus increasing the free carrier density. Initially demonstrated for ZnO NCs,^34,153^ photodoping was later extended to degenerately doped MO NCs such as ITO (Figure 6d).^8^ Schimpf et al. demonstrated postsynthetic tuning of the electron density in ITO via photodoping, independently of Sn content. Notably, the number of photoinjected electrons does not depend on the carrier density introduced by aliovalent doping, consistent with stabilization of photogenerated electrons by the Sn-doped In_2_O_3_ lattice.^8^ The Authors also emphasized that photoinjected electrons can be quantitatively removed via oxidative titration, which makes it possible to disentangle how the LSPR energy depends on Sn composition from its dependence on electron density. Importantly, photodoping enables accumulation of tens to hundreds of delocalized electrons per nanocrystal, which can subsequently be extracted through controlled oxidative titration. Spectroscopic studies with molecular electron acceptors such as 2,3,5,6-Tetrafluoro-7,7,8,8-tetracyanoquinodimethane (F4TCNQ) further demonstrate that photodoped ITO NCs can sustain multielectron transfer, including two-electron reduction of a single acceptor molecule, revealing exceptionally high charge-storage densities and chemically addressable electron reservoirs.^154^

**Figure 6:**
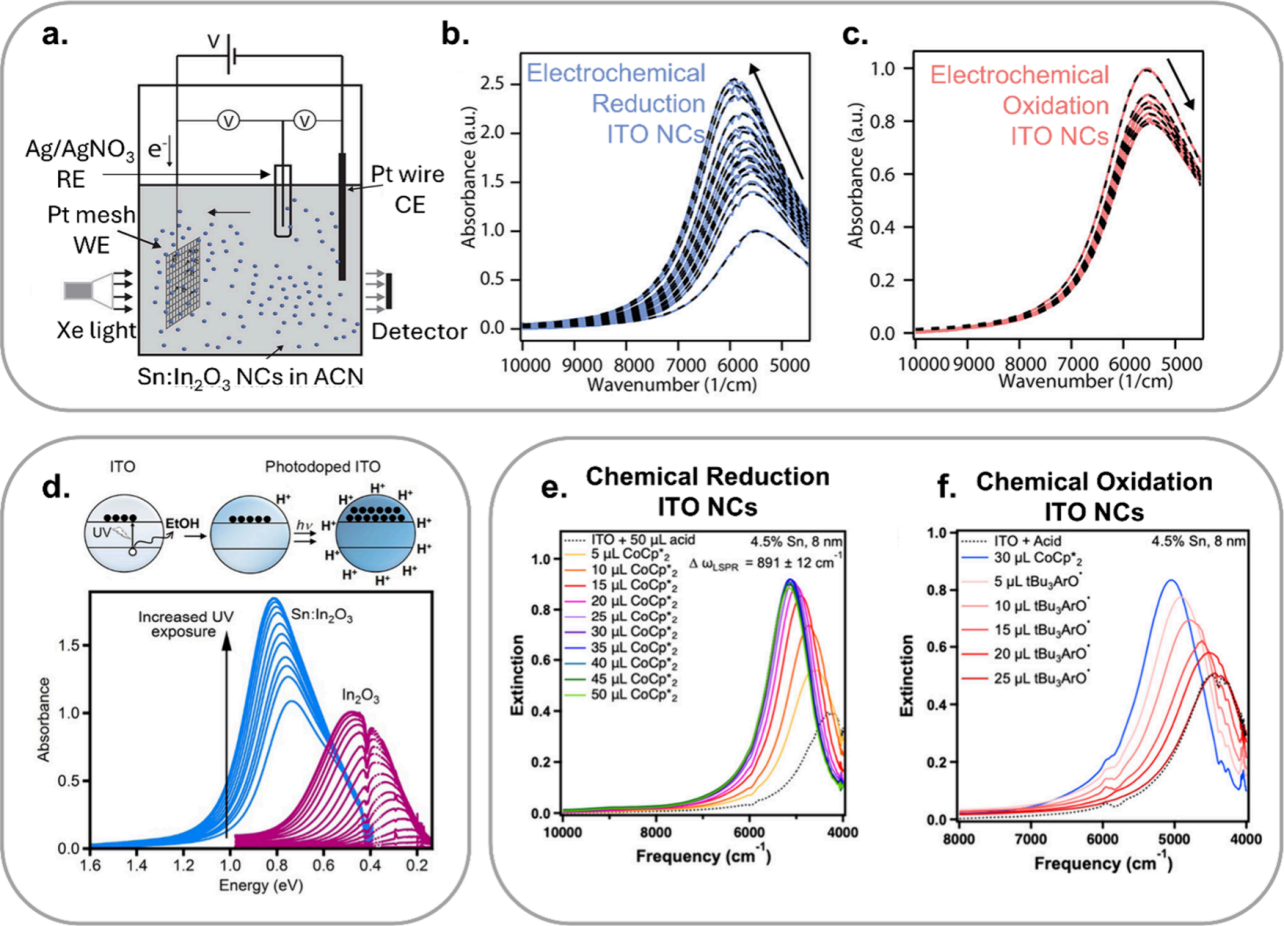
Overview of ITO nanocrystals LSPR postsynthesis modulation. (a–c) Spectro-electrochemical tuning of the infrared LSPR in ITO nanocrystals. (a) Schematic of the in situ spectro-electrochemical cell used to record IR absorbance of ITO NCs under applied bias, (b) Stepwise reduction leads to a blueshift and increase of the LSPR absorbance, whereas (c) oxidation reverses the process. (d) UV exposure induced LSPR tuning in In_2_O_3_ and ITO nanocrystals. Band-gap excitation in the presence of a sacrificial hole scavenger drives electron accumulation in the nanocrystals with charge balance maintained by H^+^ counterions provided by the oxidation of a sacrificial hole-scavenger (e.g., EtOH). (e, f) Effect of chemical reduction and oxidation on the LSPR position and strength of ITO nanocrystals. (e) Chemical reduction is obtained by volumetric addition of decamethylcobaltocene (CoCp*_2_) to an acidic solution of ITO nanocrystals leading to a blueshift and increase of the ITO nanocrystals LSPR absorbance. (f) After chemical reduction, the addition of a mild oxidant 2,4,6-Tri*tert*-butyl-phenoxy radical (tBu_3_ArO*) restores the initial LSPR position – black dotted line. Panels (a–c) adapted with permission from ref 151. Copyright 2018, American Chemical Society. Panel (d) adapted with permission from ref 8. Copyright 2015, American Chemical Society. Panels (e) and (f) are adapted with permission from ref 152. Copyright 2022, American Chemical Society.

Beyond electrochemical modulation and photodoping, reductive chemical titration offers a third route to postsynthetic LSPR control. In this approach, a molecular reductant is added in stepwise aliquots to inject electrons while monitoring the extinction spectrum. Tandon et al.^152^ used decamethylcobaltocene (*CoCp* *_2_ *)* titration to chemically reduce colloidal ITO NCs (Figure 6e) across systematic series in diameter and Sn doping.^91^ Moreover, the chemically injected electron density was observed to saturate. In the excess-reactant regime, the saturation level does not vary with Sn% or nanocrystal diameter and is determined only by the Sn concentration set during synthesis. Adding an oxidant (e.g., 2,4,6-tri*tert*-butyl-phenoxy radical) fully recovers the initial LSPR energy (Figure 6f), supporting an interpretation in terms of reversible changes in electron density.

Finally, surface ligands in colloidal doped MO NCs enable controlled synthesis and colloidal stability. Moreover, they also influence near-surface electronic structure, interparticle coupling, and overall functional performance. Beyond ligand removal, postsynthetic ligand exchange with dipolar molecules provides a direct route to tune surface electrostatics. Electron-donating and electron-withdrawing ligands can form interfacial dipole layers and shift the work function in ways that cannot be achieved by adjusting dopant concentration alone.^155^ Consistently, changes in the LSPR upon ligand exchange have been reported across a range of doped MO NC systems. The mechanistic basis of these optical changes has been clarified by electronic-structure analyses that explicitly include dopants and surface states, which point to carrier depletion near the nanocrystal surface.^7,151^

### 4.4 Depletion Layer in Doped Metal Oxide Nanocrystals

It is widely recognized that the carrier-density profile is not spatially homogeneous even in nominally homogeneously doped nanocrystals. This internal inhomogeneity can give rise to asymmetric LSPR line shapes and to the emergence of multiple plasmon modes, even in spherical nanocrystals with uniform dopant distributions (i.e., without a chemically defined core/shell structure).^7,148,151,156^ As in conventional semiconductors, the presence of a surface introduces defect states that can localize carriers and thereby generate a space-charge region, commonly referred to as a depletion layer (DL).^151,157^ In n-type, degenerately doped MO NCs such as ITO, surface defect states often behave as acceptors. Their occupation produces a negatively charged surface that is compensated by a positively charged space-charge region in the interior, yielding upward band bending (Figure 7a,b). Depletion therefore corresponds to a smooth reduction of the free-electron concentration near the surface. This leads to a radially graded carrier-density profile and highlights the limitations of the flat-band, spatially homogeneous approximation commonly adopted in the simplified Drude description (Figure 7a,b).^7,148,151,156^ Once band bending is introduced, a key parameter becomes the depletion-layer width, which is set by the electrostatic mismatch between the surface and the bulk potentials. Moreover, Fermi-level pinning at surface defect states determines the surface potential, whereas dopant placement and activation determine the bulk potential. Solving Poisson’s equation in spherical coordinates provides a quantitative relationship among depletion width, surface potential and charge density, enabling calculation of the carrier-density profile throughout the NC (see Section 4.4).^7^

**Figure 7:**
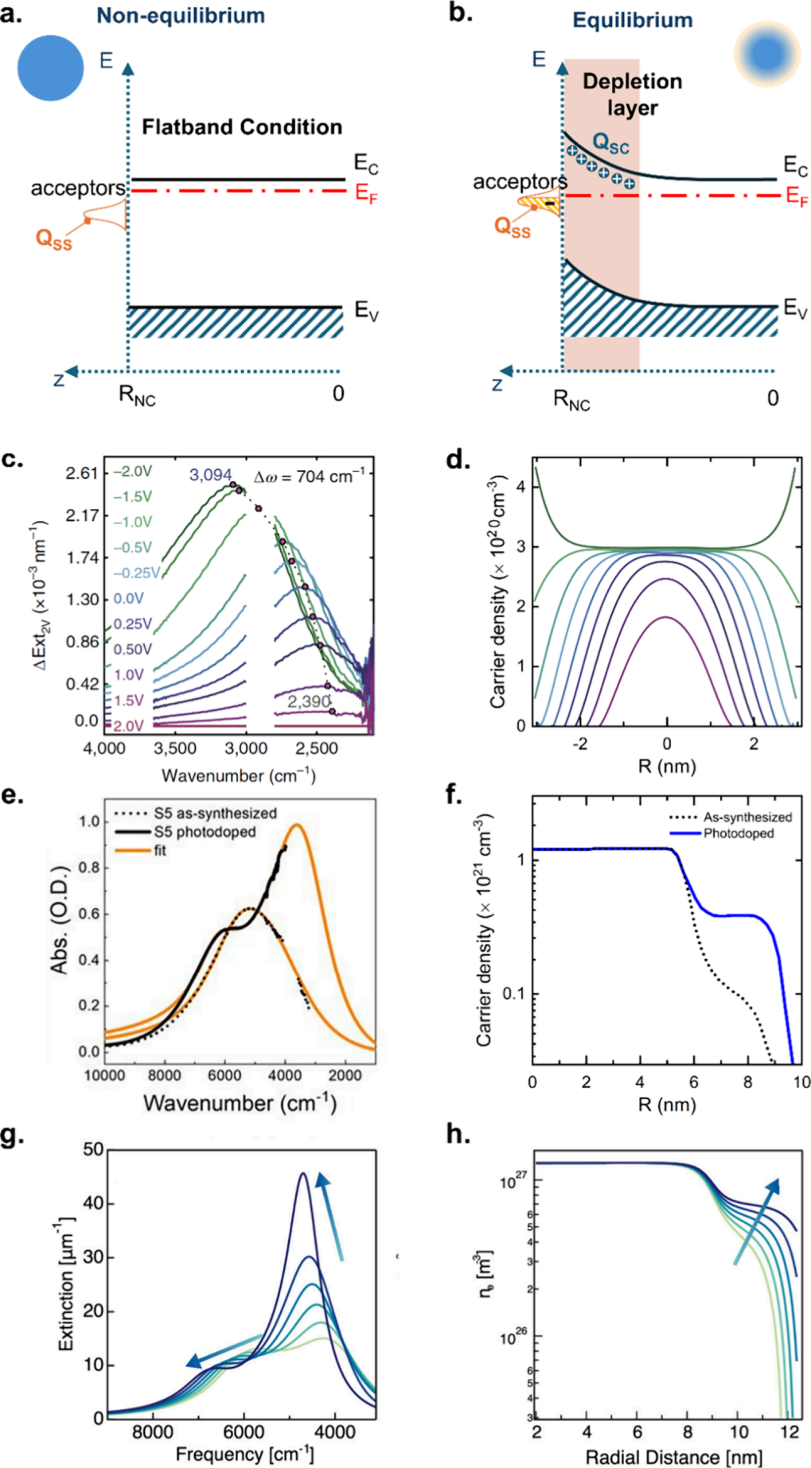
Carrier-density profiles and depletion-layer modulation governing the optical response of doped metal oxide nanocrystals under postsynthetic modulation (electrochemical charging, photodoping, and chemical titration). (a) Nonequilibrium flat-band approximation commonly used for metal nanocrystals. (b) Band profile of an n-type semiconductor nanocrystal with acceptor-like surface states. Occupation of surface states generates surface charges (Q_SS_) compensated by the space-charge region (Q_SC_), producing a surface depletion layer (shaded orange region). E_C_, E_S_, and E_F_ denote the conduction band, valence band, and Fermi level, respectively. In core/shell architectures, additional interfaces give rise to more complex band profiles, including double band bending. (c) LSPR spectral evolution of 1%-7.4 nm ITO nanocrystal films under electrochemical modulation, shown as extinction changes relative to 2.0 V (ΔExt_2 V_). (d) Corresponding simulated radial carrier density profiles. (e) LSPR spectra of ITO/In_2_O_3_ core/shell nanocrystals before and after photodoping and (f) simulated carrier-density profiles from fitting models. The emergence of dual plasmon modes after photodoping is correlated with carrier accumulation in the shell and modulation of the depletion layer. (g) Simulated LSPR extinction spectra and (h) radial carrier-density profiles with electron transfer from CoCp*_2_ raises the Fermi level, compressing the surface depletion layer and increasing carrier density in the optically active shell, leading to uniform carrier distribution and modification of the LSPR from a multimode response to a single higher-intensity peak as the core and shell modes progressively merge. Panels (c) and (d) adapted with permission from ref 7. Copyright 2018, Springer Nature. This material is not included under the Creative Commons license of this article and remains subject to third-party rights. Panels (e) and (f) adapted with permission from ref 97. Copyright 2022. The Authors under a Creative Commons CC BY 4.0 License, published by Springer Nature. Panels (g) and (h) adapted with permission from ref 158. Copyright 2023, American Chemical Society.

Lounis and co-workers provided early direct evidence for depletion regions in surface-segregated MO NCs. They distinguished *chemically depleted shells*, arising from undoped or unactivated dopants at the surface, from *electronic depletion layers* that originate from band bending when regions with different carrier concentration coexist.^6^ Although depletion-layer formation is well-established at bulk semiconductor interfaces and in thin films, it was long underappreciated in nanocrystals, especially in noble metals nanoparticles. This is largely because the very high free-carrier density in noble-metals leads to strong electrostatic screening, and the comparatively low density of electronically active defects make a depletion layer of appreciable width unlikely to form.

Zandi and co-workers later experimentally investigated how differences between surface and bulk energies modulate depletion and, in turn, the effective dielectric response and the LSPR line shape.^109^ They introduced a quantitative framework in which the corresponding carrier-density profile is computed, providing a direct route to rationalize the LSPR shifts observed under electrochemical charging. As a natural extension, deliberate engineering of the carrier-density profile emerges as a powerful tool for tuning not only the plasmonic response but also the depletion-layer width, and consequently the sensitivity to the external environment. In this context, continuous growth synthesis has emerged as the most suitable strategy to intentionally engineer the depletion layer width by tailoring the dopant placement within nanocrystals. Jansons et al.^12^ and Crockett et al.^96^ used slow-injection growth to impose controlled radial dopant gradients in ITO NCs, including core-doped, shell-doped, and uniformly doped structures at constant overall Sn content. They showed that the associated changes in depleted-volume fraction strongly affect LSPR energy, mode splitting, and dielectric sensitivity. Building on this, Gibbs et al.^109^ demonstrated that continuous growth can produce nanocrystals with distinct core and shell regions, that yield dual-mode LSPR peaks associated with each region. They further showed that placing the higher carrier concentration in the core or in the shell leads to markedly different LSPR line shape and spectral evolution. This behavior is consistent with band bending and with the presence of a surface depletion layer which depends on the internal carrier density profile. Modulating dopant placement through continuous growth, shifts plasmon modes and changes their relative intensities, while quantitative interpretation typically requires fitting or simulation approaches that explicitly capture the internal carrier distribution (see Section 4.4).

Using such approaches, recent work has demonstrated that depletion-layer modulation is essential not only for explaining spectral features that cannot be captured by homogeneous-core Drude models^7,148,151,156^ but also to rationalize and deliberately tune the LSPR under postsynthetic charge modulation by electrochemical, photochemical or chemical approaches (Figure 7c–h).^91,97,159–161^ Charge injection compensates the depleted region, expands the optically active plasmonic core/shell, and increases the LSPR intensity with only a modest blue-shift. This trend cannot be explained simply by a uniform increase in carrier density, which would induce a stronger blue shift and would miss the coupled changes in intensity and spectral shape observed experimentally. For example, the optical response measured under electrochemical charging can be reproduced only within a depletion-layer framework in which injected carriers fill the depleted region (Figure 7c,d).^7,151^ The resulting tuning capabilities, reflected in coupled changes in resonance intensity and peak position (Figure 7c), highlights the central role of surface energy, which depends on both doping level and the nanocrystal size. Simulated carrier-density profiles that evolve with electrochemical potential through changes in surface energy and depletion-layer width reproduce the experimental observations (Figure 7d).

An analogous framework has been applied to photodoping. Ghini et al.^97^ investigated ITO nanocrystals and ITO/In_2_O_3_ core–shell architectures and showed that the photodoping response is best understood when band bending and the associated depletion layer are included. Features such as LSPR peak splitting (Figure 7e) arise because photodoping primarily modifies the depletion-layer width, with photogenerated electrons preferentially filling depleted regions. In series of ITO nanocrystals with increasing In_2_O_3_ shell thickness, photodoping mainly reshapes the depletion region and band-bending in the shell rather than increasing the dopant density in the core region (Figure 7f). This effectively expands the “active” plasmonic volume without substantially raising the core carrier density, leading to strongly size- and architecture-dependent optical responses, including red shifts and peak splitting that deviate from the simple blue shift expected for a uniform Fermi-level rise.^97^ Similarly, in *CoCp* *_2_ reductive titrations of dopant-segregated ITO, Tandon et al.^158^ showed that the full evolution of LSPR energy and extinction in core-doped nanocrystals requires changes in both the surface Fermi level and the shell donor concentration (Figure 7g). Chemical reduction progressively fills the surface depletion layer while increasing carrier density primarily near the surface. The magnitude of LSPR modulation therefore depends on the initially depleted volume fraction, leading to strong size- and dopant-dependent responses (Figure 7h). From a near-field perspective, reduction in core-doped nanocrystals effectively creates a new conducting shell where the optical field is strongest, driving field redistribution. In shell-doped nanocrystals, reduction instead produces a comparatively smaller enhancement because the near-surface region is already plasmonic.

Overall, these postsynthetic strategies highlight the depletion layer as a primary lever for LSPR tunability in doped MO NCs. Electrochemical charging and photodoping mainly act by filling (or restoring) the surface-depleted region, shifting the surface potential and changing the active plasmonic volume within an otherwise fixed dopant landscape. As a consequence, even at identical nominal dopant concentrations, distinct radial dopant distributions can yield markedly different plasmonic, photoelectrochemical and charge-related properties, because they reshape the fraction of nanocrystal volume affected by depletion and regulate surface-mediated charge exchange processes. Quantitative description of these effects requires theoretical modeling that moves beyond homogeneous approximations. Accurate interpretation of the experimentally observed spectral and transport modifications therefore relies on appropriate electromagnetic and electronic modeling frameworks explicitly accounting for spatial variations in carrier density on the nanoscale induced by depletion.

### 4.5 Modeling the Optical Response of Doped Metal Oxide Nanocrystals

As discussed in the previous sections, the precise dopant control enabled by continuous-growth synthesis calls for an equally sensitive evaluation method, a role that the LSPR can fulfill. Interpreting LSPR shifts, line shape and line widths that originate from dopant placement, surface depletion, Fermi-level pinning, or postsynthetic modification requires detailed electromagnetic modeling of the nanocrystals. In the classical description of metal nanoparticles, photoexcitation drives a collective oscillation of a bulk like and spatially uniform electron gas.^162,163^ By contrast optical modeling of degenerately doped MO NCs must incorporate, within the Mie approach, the spatially varying carrier density profile that arises from Fermi level pinning, carrier depletion and dopant placement. This yields a richer plasmonic response and makes doped MO NCs fundamentally different from noble metals. The underlying principles of the plasmonic response of metallic nanoparticles is based on the seminal work of Gustav Mie, who first solved the scattering problem for a spherical particle under an incident electric field and obtained exact solutions describing its multipolar modes.^110,162,163^ For a given material dielectric function, Mie theory connects the dielectric response to the frequency-dependent polarizability α­(ω) of a sphere embedded in a dielectric medium. Within this framework, nanoparticles are commonly treated as homogeneous spheres, or ellipsoids in the Mie-Gans generalization, and their dielectric function is modeled by a Drude term:^163^

$$\varepsilon \left(\omega \right)=\varepsilon_{\infty }-\frac{\omega_{p}^{2}}{\omega^{2}+i\gamma \omega }$$

where ε_∞_ accounts for high-frequency interband contributions not accounted by the Drude term, ω is the incident wavelength angular velocity (ω = 2π · *c*/λ), ω_*p*_ is the bulk plasma frequency determined by the free-electron density *n*_*e*_ and the effective electron mass *m*_e_^*^ as $\omega_{p}^{2}\propto \frac{n_{e}}{m_{e}^{*}}$ , and γ is the damping rate, often treated as frequency independent and occasionally modified to include size-dependent scattering processes.^6^ Under the quasi-static approximation, valid for sufficiently small and well-separated nanoparticles, the dipolar mode dominates the response. In this limit, the LSPR condition follows the Fröhlich criterion, which predicts a maximum extinction when:

Re{ε(ω)}=−2εm

for a spherical nanoparticle embedded in a medium of dielectric constant ε_*m*_.

As discussed previously, MO NCs are often analyzed using the same formalism by approximating them as homogeneous particles with a Drude-like dielectric function, which implicitly assumes a spatially uniform carrier density. While this approximation may hold for noble metals, it becomes insufficient for MO NCs when band bending is present. In that case, assuming a uniform *n*_*e*_ does not capture key LSPR features. This limitation was highlighted even for nominally homogeneous MO NCs by Lounis et al.^6^, who showed that incomplete dopant activation near the surface produces spectral features incompatible with a simple Drude model. They attempted to address this by introducing a phenomenological energy-dependent damping term, but this strategy does not explicitly incorporate depletion-layer effects or spatial variations in carrier concentration.^6^

A more rigorous treatment was introduced by Zandi et al.,^7^ who explicitly calculated the radial carrier-density profile by solving the Poisson equation under surface Fermi-level pinning condition (Figure 8a). The band potential Φ­(r) satisfies:

$$\frac{\partial^{2}\Phi }{\partial r^{2}}=-\frac{\rho \left(r\right)}{\varepsilon_{s}\varepsilon_{0}}$$

where ρ­(r) includes contributions from free carriers and ionized dopants, and ε_*s*_ and ε_0_ are the static dielectric constant (usually approximated as ε_∞_) and vacuum permittivity, respectively. This approach allows one to assign, at each point of the nanocrystal, a specific dielectric function described by the Drude model, and through finite element modeling (FEM) simulation, to calculate the absorption cross-section.^161^ To reduce computational complexity, Zandi et al. approximated the NC as a core surrounded by concentric shells, each characterized by an averaged carrier density and a corresponding dielectric function, thereby discretizing continuous profile (Figure 8b). Effective-medium mixing formalisms such as Maxwell Garnett, Bruggeman, Reich and Shklovskii^165^ can then be used to combine contributions from adjacent layers, depending on the relative magnitude of dielectric functions, surrounding medium, and the nanocrystals separation.

**Figure 8:**
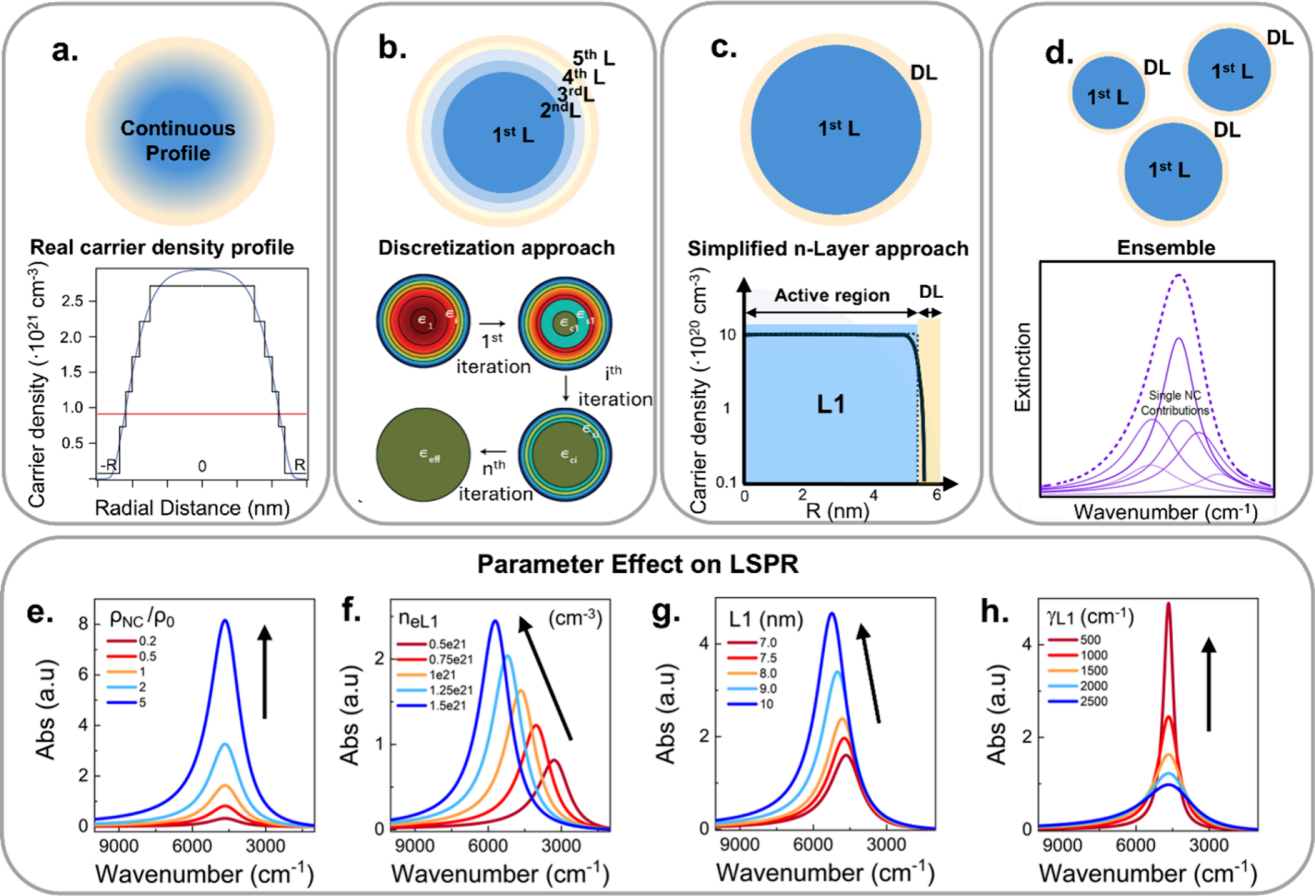
Modeling approaches for describing the spatial carrier-density profiles used to calculate the corresponding LSPR in the case of homogeneously doped ITO nanocrystals. (a) Schematic illustration of a generic radial carrier-density profile. The varying profile is associated with surface band bending and results in surface depletion. (b) Discretization into step-like functions of the previous continuous carrier density profile represented as concentric shell layers. Workflow for calculating the effective dielectric function by combining the dielectric responses of the individual shells. (c) Minimal two-layer representation (core and depletion layer) for the previously depicted carrier density profile. (d) Heterogeneous ensemble Drude approximation (HEDA) approach incorporates the minimalistic approach shown accounting for the polydispersity of the nanocrystals (in terms of radius and inner carrier density). (e–h) LSPR modulation associated with discrete variations in model parameters for a core-depletion ITO nanocrystal structure: (e) nanocrystals concentration in the solution, (f) core carrier density, (g) radial extent of the core active region within the nanocrystal and (h) damping parameter. Panels (a) and (b) adapted with permission from ref 7. Copyright 2018, Springer Nature. This material is not included under the Creative Commons license of this article and remains subject to third-party rights. Panel (d) adapted with permission from ref 164. Copyright 2020, American Chemical Society. Panels (c) and (e–h) adapted with permission from ref 156. Copyright 2023, The Authors under a Creative Commons CC BY 4.0 License, published by American Chemical Society.

For high dielectric constant materials, like ITO NCs in a solvent environment, the Maxwell-Garnett approximation is appropriate. For a generic system composed of material A embedded in material B, with dielectric constants ε_*A*_ and ε_*B*_, the effective dielectric function ε_*eff*_ can be determined as

$$\varepsilon_{eff}=\varepsilon_{B}\frac{\left(\varepsilon_{A}+2\varepsilon_{B}\right)+2F\left(\varepsilon_{A}-\varepsilon_{B}\right)}{\left(\varepsilon_{A}+2\varepsilon_{B}\right)-F\left(\varepsilon_{A}-\varepsilon_{B}\right)}$$

with $F=\frac{V_{A}}{V_{A}+V_{B}}$ the volume fraction of material A. This expression can be applied by taking A and B as the core and shell in a core/shell system, or by taking A as the NC and B as the surrounding medium. Since ε_*A*_ and ε_*B*_ are in general complex numbers, ε_*eff*_ is a complex number as well, with nontrivial real and imaginary parts. This makes it important to represent the carrier density profile with the minimum number of layers needed. Each Drude-like layer introduces at least three parameters, including carrier density, damping parameter and volume fraction. When fitting experimental LSPR spectra, overparameterization becomes a primary concern, so the layered representation should remain minimal while still capturing the relevant physics. Petrini et al.^156^ demonstrated that a two-layer model is sufficient for homogeneous nanocrystals (Figure 8c), accounting for the core and the depletion layer at the surface, while a three-layer model is required for core/shell nanocrystals to represent the core dielectric function, the shell, and the surface depletion region. In this refined picture, the damping parameter γ is no longer treated as an ad hoc energy-dependent term, but is instead linked to physical quantities such as bulk electrons mean free path, the electronic active region and their interplay^164,166^

$$\gamma =\frac{{\left(3\pi^{2}\right)}^{1/3}\hbar }{m_{e}^{*}}n_{e}^{1/3}\left(\frac{1}{R_{active}}+\frac{1}{l_{\mathrm{bulk}}}\right)$$

where *R*_*active*_ is the optical active region of the nanocrystal, i.e. the nominal radius of the nanocrystal minus the depleted shell thickness, and *l*_*bulk*_ is the bulk electrons mean free path. This expression also makes explicit that the damping γ depends on carrier density *n*_*e*_, and is therefore governed by underlying physical parameters. Consequently, changes in *n*_*e*_, whether set during synthesis or induced by external modulation, renormalize γ and preclude treating it as an independent parameter.

To further refine this picture, inhomogeneity between nanocrystal sizes and carrier density profiles are considered. Gibbs et al.^164^ introduced the heterogeneous ensemble Drude approximation (HEDA), which incorporates experimentally measured size distributions and assumes a Gaussian distribution of carrier densities (Figure 8d). By summing the optical contributions over the measured size distribution, the HEDA model provides highly accurate predictions of experimental LSPR spectra. Once ε_*eff*_(ω) of the NC, or its layered representation, is obtained, the optical response can be computed directly. For small, well-separated spherical nanocrystals in the quasi-static regime, and with negligible scattering, the absorption cross-section σ_*abs*_(ω) is given by the imaginary part of the polarizability α­(ω):

σabs(ω)=kIm{α(ω)},k=ω·εmc

where ε_*m*_ is the dielectric constant of the medium. The polarizability of a sphere of radius *R* embedded in a medium of dielectric constant ε_*m*_ is ^162^:

$$\alpha \left(\omega \right)=4\pi R^{3}\frac{\varepsilon_{\mathrm{eff}}\left(\omega \right)-\varepsilon_{m}}{\varepsilon_{\mathrm{eff}}\left(\omega \right)+2\varepsilon_{m}}$$

Finally, the absorbance *A*(ω) of a dispersion of nanocrystals could be obtained via Beer–Lambert law, as

$$A\left(\omega \right)=\frac{N\sigma_{\mathrm{abs}}\left(\omega \right)L}{\ln\left(10\right)}$$

where *N* is the nanocrystal concentration (number of nanocrystals for unit volume) and *L* the optical path length.

Overall, the workflow links the Poisson equation solution to obtain the carrier-density profile to a layered effective dielectric function and the resulting polarizability, enabling a direct prediction of the LSPR line shape in the absorbance spectrum. This procedure provides detailed physical insight into how dopant distribution, surface depletion, and ensemble heterogeneity collectively shape the observed optical response. Moreover, this approach enables the identification of specific parameters affecting the plasmonic response. Figure 8e–h illustrates how, for an ITO NCs dispersion with a homogeneous core and a depletion layer, changes in nanocrystal concentration, core carrier density, depletion-layer extent, and damping parameter γ independently affect the LSPR response. Finally, it is worth noting that the LSPR can also be modulated by a parameter that is often overlooked, namely the effective electron mass *m*_e_^*^. Magnetic circular dichroism (MCD) has been used to decouple *n*_*e*_ from *m*_e_^*^, revealing that in ITO NCs the effective mass is not constant but varies systematically with doping level, deviating from the bulk value.^119^ This introduces an additional variable that must be considered when comparing samples synthesized with different doping levels, whereas it is not expected to be a dominant factor when comparing syntheses with similar dopant concentrations.

Taken together, these results show that modeling provides both qualitative and quantitative insight into plasmonic behavior of doped MO NCs. LSPR modulation does not depend on a single parameter, but instead emerges from the interplay of multiple, independently tunable variables, including carrier density and its spatial distribution. Many of these quantities, such as dopant distribution, depletion-layer extent, and even *m*_e_^*^, are not merely fitting parameters. They are encoded during nanocrystals synthesis and can also be tuned postsynthetically, as discussed in the previous sections. This establishes a direct link between predictive modeling and programmable synthesis routes, enabling the engineering of optoelectronic response through control of nanoscale architecture and dopant placement.

## 5 From Programmable Synthesis toward Applications

Continuous growth links synthetic programmability to application-level programmability by providing a unifying route to connect dopant profiles, surface chemistry, and nanocrystal architectures with targeted functional outcomes in optical, electronic, catalytic, electrochemical, sensing and biomedical applications. In doped metal oxide nanocrystals, these parameters govern key responses including LSPR properties, charge transport, catalysis electrochemical behavior and interfacial reactivity. By shaping radial dopant profiles, such as core-doped, shell-doped, or graded architectures, the electronic structure can be engineered within single nanocrystals. This level of control directly governs how charges are generated, separated, transported, and stored, and it opens routes to multifunctional and stimuli-responsive materials across a broad range of optoelectronic technologies. Although doped MO NCs are already established in energy-related applications, controlled fabrication methods can still unlock additional value in two ways. First, precise design of the building block supports ad hoc and fine-tuned systems, which can reduce waste in both materials and energy consumption. Second, nanocrystals designed with a specific energy profile on demand can better meet strict requirements on combined properties and can be adapted more easily as application constraints and socio-economic dynamics evolve. Accordingly, the following discussion also considers general properties of metal oxide that can be enhanced through continuous-growth approaches and translated across different application areas.

### 5.1 From Solutions to Nanocrystal Thin Films

Most applications require translating programmable nanocrystal properties from colloidal dispersions to solid-state platforms, typically by processing them into thin films. Solution-based deposition techniques such as spin coating, dip coating, self-assembly, and inkjet printing provide scalable fabrication routes with precise thickness control.^167,168^ However, the step from dispersion to functional thin film is a critical bottleneck in translating nanoscale design into macroscopic optoelectronic performance. Colloidal synthesis offers excellent control over composition, size, dopant placement, and electronic structure at the single-particle level, but these advantages can be compromised during film formation. Preserving and rationally integrating the optical and electrical properties of individual nanocrystals in a continuous, device-relevant solid therefore requires careful control over film quality, interparticle coupling, and light-matter interaction across length scales. Multiscale optical modeling has shown that nanocrystal-nanocrystal interactions and substrate effects can produce measurable deviations in LSPR behavior relative to colloidal dispersions.^9^ In thin films, thickness, packing density, surface roughness, and refractive index contrast influence absorption, scattering, interference, and optical confinement. In systems where plasmonic responses are central, even subtle variations in nanocrystal spacing or local dielectric environment can lead to pronounced changes in resonance energy, line width, and modulation depth. Uniformity across the film is therefore fundamental to avoid inhomogeneous broadening and spatially varying optical responses that degrade device performance, particularly in large-area architectures.

Electrical transport is equally sensitive to the details of film formation. Charge carrier mobility in nanocrystals is governed by interparticle contacts, ligand chemistry, and percolation pathways rather than by intrinsic band structure alone. Processing steps such as ligand exchange, annealing, or sintering must be optimized to enhance electronic coupling without erasing nanoscale features that underpin optical functionality, such as quantum confinement or radially engineered dopant profiles. In optically active films, this balance is delicate. In fact, excessive densification may improve conductivity but can increase optical losses, while insufficient coupling can preserve optical signatures at the expense of slow or inefficient electrical response. In this context, carrier placement control can be quite valuable. Experiments on ITO NCs films demonstrated that dopant-enriched surfaces lead to improved conductivity through a reduced surface depletion width.^106,169,170^ Variable-temperature conductivity further correlated increased electron localization lengths with surface dopant density, confirming that radial dopant profiles directly impact charge transport.^169^ Ligand exchange represents another critical tool in bridging synthesis and application.^171^

Moving closer to applications, charge transport in these assemblies must be understood with explicit consideration of the carrier-density profile within individual nanocrystals, which becomes especially relevant when the carrier-density profile is intentionally controlled synthetically. It also requires an understanding of how charge is transferred across nanocrystal networks. As discussed above, continuous growth synthesis enables control over carrier density that is central for tuning the electrical conductivity of MO NC films. Because electron transport in nanocrystal networks proceeds primarily via hopping between localized states associated with individual nanocrystals, modulation of depletion width and dopant gradients has profound consequences for charge mobility. Film conduction can be described within disordered media frameworks, for instance by modeling the NC film as a random resistance network as implemented in the Miller and Abrahams model.^172^ In NC assemblies, the spatial localization of particles and random interparticle barriers shift hopping-controlled mechanisms to significantly higher temperatures, often approaching room temperature. Continuous-growth effectively tunes both localization length and the interparticle tunneling barrier, enabling transitions between conduction regimes, that include Efros-Shklovskii variable-range hopping (ES-VRH), Mott VRH, nearest-neighbor hopping (NNH), and granular metallic conduction.^173–175^ These regimes share a general temperature dependence of conductivity defined by a material-dependent parameter (*T*_0_) and an exponential β that depends on the transport mechanism. Typical values are VRH = 1/4 (three-dimensional transport) or VRH= 1/3 (two-dimensional transport), ES-VRH = 1/2, and NNH = 1.^176,177^ Temperature-dependent conductivity measurements allow identification of the operative mechanism, and Zabrodskii analysis provides a practical route to extract both β and *T*_0_ through linear fitting.^178^ Moreover, Chen et al. recently showed that NC films exhibit an insulator–metal transition (IMT) analogous to the Mott transition, described by carrier density, the valley degeneracy of the conduction-band minima, and the electronic contact radius between nanocrystals (ρ_*c*_). For touching spheres, ρ_*c*_ is defined by the interparticle contact geometry. Depending on whether the interface is facet-to-facet (a-contact) or off-facet (b-contact), the contact radius changes and the critical carrier density for the IMT changes accordingly.^179^ When an insulating shell separates adjacent nanocrystals, the criterion is further modified by shell thickness and the b-contact radius. Three scenarios may then arise. One is intrinsically insulating nanocrystals that fail the classical Mott criterion. The second is metallic nanocrystals arranged in an insulating network when the Chen IMT criterion is not satisfied. The third is a metallic percolating network. Multiple studies show that dopant segregation toward the NC surface increases the electronic contact radius, thereby lowering the carrier density required to cross the IMT. This effect becomes stronger when combined with postdeposition treatments such as ligand stripping or ALD infilling.^7,96,106^ Continuous-growth methods therefore provide a powerful synthetic way to engineer the percolation landscape governing long-range charge transport in disordered NC films. By minimizing dopant heterogeneity, reducing energetic disorder, and controlling radial carrier distributions, continuous growth also enables more predictable transitions between insulating and metallic regimes, with conductivity spanning orders of magnitude. These trends have been demonstrated across several MO NC systems, including In_2_O_3_, ZnO, and TiO_2_.^169,170^

Staller et al. further confirmed that synthetic control over depletion-layer width directly impacts film conductivity.^106^ Using nanocrystals with identical overall dopant concentrations but different dopant profiles (core-enriched versus shell-enriched), they showed that increasing the dopant concentration toward the surface shrinks the depletion region and increases the effective contact radius, thereby enhancing conductivity. In core-enriched nanocrystals, conductivity rises as core radius increases, reducing the fractional depletion volume. Additional conductivity enhancements were obtained by tuning Fermi-level pinning through alumina capping, which reduces depletion width. Comparable results have been obtained by physically growing shells on doped nanocrystals^179^ as well as through sintering and postsynthetic photodoping treatments, as reported by Greenberg et al. and Benton et al.^169,180,181^ Band-bending calculations and temperature-dependent extractions of the localization length corroborate that conductivity changes correlate directly with depletion-layer modulation, consistent with hopping and granular-conduction frameworks. In a subsequent study, Staller et al. demonstrated that transport in doped ITO NC films is governed by the interparticle contact conductance, which determines whether the assembly lies in the insulating or metallic regime.^182^

Ultimately, moving from colloidal particles to thin films is not simply a deposition step. It is a materials engineering problem in which performance emerges from the collective response of the film rather than from isolated particles. Success depends on strategies that translate nanoscale control into mesoscale order while preserving the designed interplay between optical absorption, carrier dynamics, and transport. A particularly relevant example of functionality emerging at the film level is gas sensing, where both optical and electrical responses are exploited. Metal oxide thin films have long been investigated for their sensitivity to gaseous environments,^183–186^ with colloidally synthesized MO NCs boosting studies on systems including ZnO,^187,188^ SnO_2_,^189^ ITO,^190^ WO_3_,^191^ and CuO,^192^ making them a well-established platforms for detecting gases including H_2_, NO_2_, CO, and O_2_. In these systems, gas adsorption-induced changes over surface charge and surface depletion layer modify both electrical conductivity and plasmonic response. More recently, attention has shifted from the intrinsic chemistry of metal oxides to the role of nanoscale design in governing sensing performance, highlighting the possibility of tuning sensitivity, selectivity, and response dynamics through controlled nanostructuring. For instance, doped ZnO nanocrystals (e.g., Ga-doped ZnO) exhibit tunable sensitivity and response times as a function of carrier density, while LSPR shifts provide a direct optical probe of gas-induced carrier modulation.^187^ Importantly, the ability to control dopant distribution and carrier density through programmable synthesis introduces an additional degree of freedom to tune sensitivity, selectivity, and operating temperature, including the possibility of efficient light-assisted sensing compared to undoped ZnO.

### 5.2 Light-Driven Dopant-Controlled MO NC Systems

Film quality becomes particularly critical when light is not only used as a probe, but directly drives functionality, as in photochromic, electrochromic, plasmonic, and photochemical systems. In this context, the solution processing of transparent conductive oxides nanoparticles with controlled optical features enables the fabrication of thin films with good optical quality and enhanced electrical conductivity.^87^ A representative optoelectronic case is the reversible modulation of optical properties under either light stimulus or an applied electrical bias, which manifests as photochromism and electrochromism, respectively.

Chromogenic materials are central to smart windows, adaptive optics, and security technologies, where controlled modulation of optical transmittance enables dynamic tuning of device transparency. In typical chromogenic metal oxides, transition-metal doping and oxygen vacancies influence chromogenic behavior and device performance, as in smart-window technologies.^193^ Typical materials platforms investigated to date include sub stoichiometric tungsten oxide (WO_3‑x_) and tungsten bronzes (M_*x*_WO_3‑x_),^35^ which combine a response in the visible spectrum (associated with intervalence charge transfer - IVCT) with a plasmonic response,^194^ and typically exhibit high coloration efficiency.^195^ Analogue photo- and electrochromic mechanisms have also been reported for molybdenum, vanadium and titanium oxides, as well as doping-dependent materials as F, In -codoped CdO (FICO).^38,196–198^

While bulk metal oxides can show pronounced chromogenic properties, nanostructured films often display improved performance, including faster switching and durability. This is generally attributed to a large active surface area and short diffusion path lengths compared with their bulk counterparts. In this sense, programmable synthesis of doped and undoped chromogenic MO NCs is beneficial because it enables control over size and crystallinity, which can maximize optical response and facilitate ion intercalation, thus improving the performance of the photoresponsive layer.^9^ Photochromic materials undergo a reversible change in optical properties, typically color or absorption spectrum, upon exposure to light. In inorganic photochromics based on doped MO NCs, photochromism is often mediated by the generation and stabilization of photogenerated charge carriers, which modify the dielectric response and the plasmonic contribution to the optical extinction. Similarly, in electrochromic devices based on plasmonic MO NCs, tuning LSPR intensity and spectral position provides a direct and quantitative control over optical modulation. Applying an external bias injects or extracts charge carriers, altering the free-carrier density and, consequently, the plasma frequency. In the context of continuous-growth synthesis and dopant placement, radial dopant engineering becomes especially relevant for dual-responsive materials whose optical response can be tailored to target the visible and the near-infrared ranges. As illustrated in Figure 9a, dopant-controlled MO NCs can be designed to achieve specific spectral responses, while electrochemical or photochemical modulation can be exploited to generate targeted coloration trends that selectively affect the visible or the near-infrared response. As discussed in Sections 4 and 5, illumination or electrochemical doping can change the free-carrier density and can redistribute carriers between core, shell, and surface regions. These changes translate into shifts of the LSPR position and into variations of resonance intensity.^7,20^ Spectral shifts often reflect spatial redistribution of carriers and local dielectric changes, while intensity modulation tracks the amount of charges stored in the system, providing a basis for constructing functional devices from multifunctional nanomaterials.

**Figure 9:**
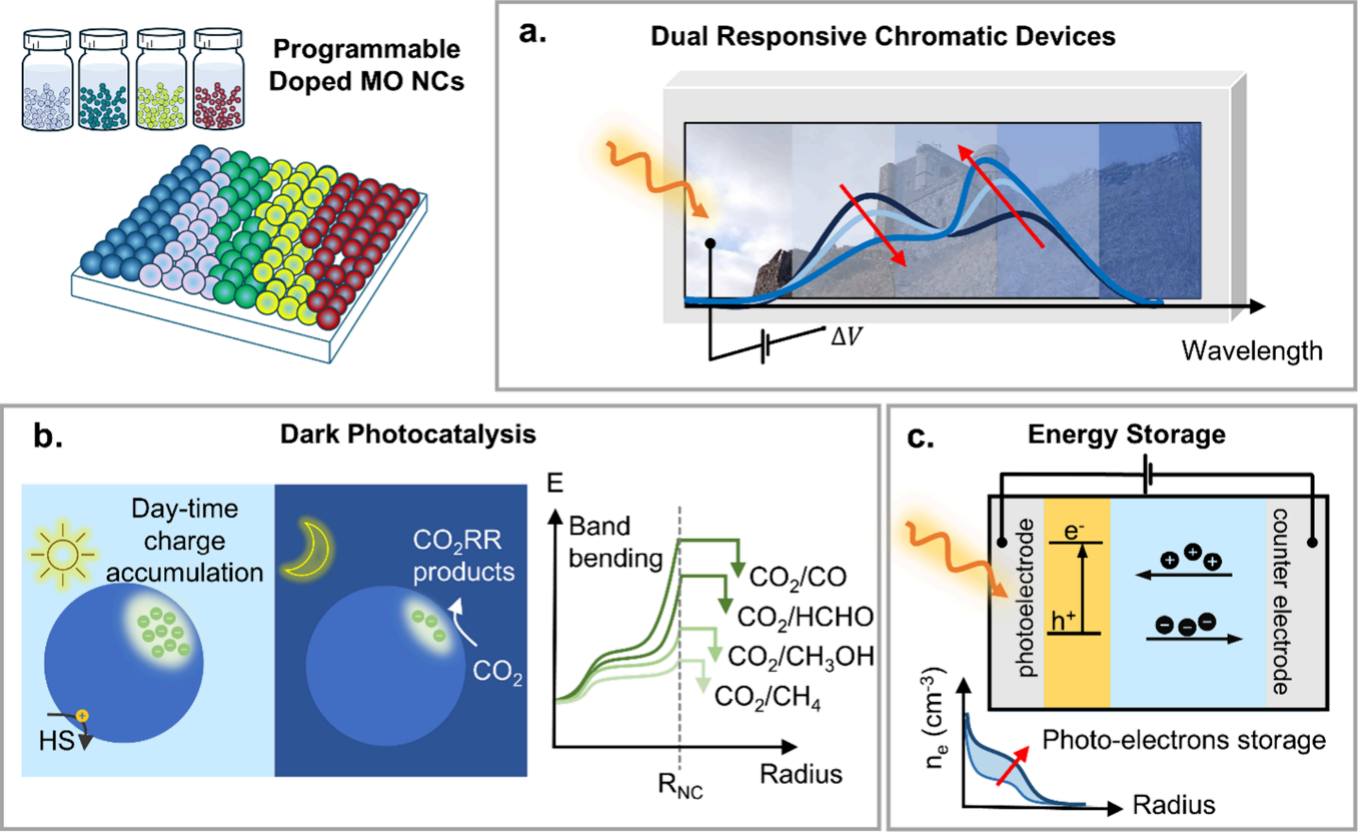
Main applicative scenarios for programmable doped metal oxide nanocrystals (MO NCs). (a) Chromogenic devices, exploiting the coloration ability of dopant-controlled MO NCs displaying dual response in the visible and near-infrared, tuned upon electrical or luminous stimuli. (b) Dark photocatalysis, i.e., on-demand release of light-induced charge accumulation for catalyzing chemical reactions during dark hours. The exploitation of band bending effects might deliver more precise selectivity toward specific chemical reactions, by designing dopant placement in the MO NCs. (c) Energy storage devices, e.g., photo batteries and photo­(super)­capacitors, where doped MO NCs would aid in integrating light harvesting and energy storage in single photoactive charge storage layers.

By engineering nanocrystal composition and dopant placement, it is possible to promote either persistent photodoping or reversible photochromic cycling. This enables optical responses that are triggered by light and retained in the dark. The same design principles extend beyond optical and electronic properties to chemical reactivity. The tunable optoelectronic features of the as-discussed materials can be exploited for the realization of materials specifically engineered for catalytic and photocatalytic processes (Figure 9b). Catalytic behavior in MO NCs depends on facet exposure, defect chemistry, and dopant location. Undercoordinated surface sites, oxygen vacancies, and aliovalent dopants have been demonstrated to lower activation barriers and stabilize key intermediates across In_2_O_3_ and related oxides.^85,95,199,200^ While such effects are typically accessed through postsynthetic modification, they point to a broader opportunity. If facets, dopants, and surface electrostatics can be programmed during growth, catalytic pathways could be designed rather than empirically optimized, directly linking synthetic control to reactivity. The benchmark materials for photocatalytic process, namely titania (TiO_2_) and zinc oxide (ZnO), are robust and cost-effective, but their wide band gaps and fast recombination rates limit solar harvesting.^201^ Such drawbacks motivate strategies that extend absorption or stabilize charges, often through heterojunction formation. In this perspective, controlled doping of MO NCs can provide tunable absorption ranges, facet control, and the introduction of active states associated with defects and heteroatoms, without necessarily relying on the formation of heterojunctions with metallic or metal oxide cocatalysts. Furthermore, tuning the conduction band edge via band bending can become pivotal for directing the selectivity of multiproduct reactions, such as CO_2_ reduction. Such reduction process has been of interest given the high value of the resulting products (methane, methanol, carbon monoxide, ethylene). Nevertheless, selectivity toward a single product is challenging because the standard reduction potentials of the competing pathways lie very close to each reduction reaction,^202^ often requiring a trade-off with catalytic activity and motivating the use of cocatalysts. The tunability of the conduction band energy provided by programmable synthesis of doped MO NCs, along with engineering of the catalyst surface and crystal facet, could be beneficial to the creation of highly selective catalytic platforms.^203^ Additionally, the compelling charge storage properties of MO NCs are accompanied by the reversibility of the photodoping process. As discussed above, ITO NCs can undergo reversible multicharge transfer through back-titration, making them suitable catalytic platform for multielectron reactions as CO_2_ reduction reaction (CO_2_RR) and multivalent or solar redox flow batteries.^8,154,204,205^

The multicharge transfer, observed in ITO, supports the formation of multielectron reduction products, such as methane (8e^–^), methanol (6e^–^) and multicarbon species.^202^ Moreover, given the ability of transferring charges in dark after photodoping, MO NCs naturally fall into the list of candidates for photodriven catalysis in the dark. This intrinsic charge storage ability of MO NCs can lead to the development of systems for dark and/or on demand photocatalysis. Dark photocatalysis relies on the concept of a material (catalyst) able to provide charges in a controlled way, triggering reactions after illumination time and detaching redox reactivity from light supply.^206,207^ To date, the focus of dark photocatalysis-related literature has pointed toward the implementation of carbon nitrides and tungsten oxide as energy storage materials applied to catalysis.^208–210^ For instance, Co_3_O_4_ demonstrate how embedding MO NCs into electrodes translates into electrochemical functionality, where reversible Co­(II)/Co­(III)/Co­(IV) redox transitions enable pseudocapacitive charge storage, highlighting that controlled nanostructuring and oxidation-state engineering can directly govern charge storage mechanisms.^211^ Engineered MO NCs could however represent a valid alternative for on-demand charge transfer, as demonstrated for instance by realizing IR-responsive carrier transfer in ITO/TiO_2_ systems, where the rational band alignment, control of LSPR and of the heterointerface enables efficient interfacial charge transfer, with hot-electron injection efficiencies up to 33% and long-lived charge separation on the microsecond time scale.^212^

Lastly, one of the most attractive yet challenging opportunities enabled by doped and undoped MO NCs is photostorage, in which photogenerated charges are accumulated and retained for subsequent energy delivery in photo batteries or photocapacitors (Figure 9c). Early studies demonstrated prolonged charge retention in colloidal nanocrystal solutions under photodoping conditions in the presence of hole scavengers. In a representative example, Brozek et al. quantified charge storage in photodoped iron-doped ZnO NCs, reporting a volumetric capacitance of 233 F per nanocrystal volume and specific capacitances of approximately 12 F g^–1^ in the dark for corresponding capacitor electrodes.^213^ Analogue charge storage properties were attested for several other doped MO solutions, namely ITO, AZO, FICO, doped and undoped WO_3_, Nb-TiO_2_ and SrTiO_3_ among the others.^8,214–216^ Despite these fundamental studies on charge retention, owing to photodoping and enhanced charge density provided by aliovalent doping and defects, the translation of such optoelectronic properties for the development of photostorage technologies is still at early stages.^61,63,95,154,216–218^ In fact, the implementation of doped MO NCs must be preceded by a careful design of the device architecture. Owing to their combined light harvesting and energy storage properties, MO NC films can be incorporated as a single layer in photo batteries and photocapacitors, overcoming the traditional combination of a solar cell and an energy storage unit (capacitor or battery) and achieving high level of integration. Most of the photocapacitor implementations often rely on four-terminal (4T) device architectures, in which light harvesting, charge separation, and storage are spatially and electrically decoupled. Typically, a photovoltaic element generates charge carriers that are routed through external circuitry into a separate capacitor or supercapacitor. Three-terminal (3T) architectures represent an intermediate step toward integration. In these devices, a photoactive electrode shares a common contact with a capacitive element, allowing partial coupling between photogeneration and storage while retaining an independent control electrode. The most integrated approach is the two-terminal (2T) photocapacitor, where photogeneration and charge storage occur within the same active layer and are addressed through a single pair of electrodes. In this configuration, illumination directly changes the charge state of the material, and the stored charge can be accessed electrochemically without intermediate components. Efficient 2T operation, however, requires materials that sustain long-lived photoinduced charges while suppressing rapid recombination, which is an intrinsic materials challenge rather than an architectural one.^219^ Here, doped MO NCs synthesized via slow-injection, continuous-growth methods provide compelling evidence that 2T photocapacitors can be feasible and potentially optimal. Radial dopant placement creates internal band bending and built-in electric fields that promote spatial separation of photogenerated carriers. These internal gradients enable nanocrystals to act as nanoscale charge-separating and charge-storing units, stabilizing electrons or holes in doped regions while isolating countercharges. Upon film assembly, the collective behavior of such nanocrystals can yield a solid able to charge with light and retain that charge in the dark. However, for such devices to reach the performance of the most common architectures presented in literature (hundreds of mF/cm^2^ or F/g), a deep investigation into both materials and device design is still required.^220–226^

## 6 Concluding Perspective

Recent progress in continuous-injection and continuous-growth syntheses has provided a practical route to connect growth kinetics, dopant incorporation, and nanoscale architecture in doped metal-oxide nanocrystals. Continuous injection converts MO NCs from static batch-synthesized products into programmable inorganic architectures. By decoupling nucleation from growth, it allows independent control of precursor flux, facet stabilization, and radial dopant placement, thereby expanding the synthetic toolbox for tailoring both structural and electronic properties. Recent works show that this synthetic method maps onto measurable outcomes. Precursor flux controls faceting and defect formation, reagent choice sets surface termination and charge localization and time-resolved dopant delivery programs carrier-density profiles that control plasmonic response, film conductivity, and electrochromic dynamics.^14,227–229^ Sequential injection schemes further enable multicomponent and core/shell structures, while temperature control stabilizes kinetic growth and supports morphology preservation.^86,95^ In this view, dopant profiles, surface chemistry, and interfaces are encoded during growth rather than imposed postsynthetically. This reduces the gap between synthesis and application because electronic structure, charge transport, and reactivity can be tuned through growth and surface processes. As both predictive control and scalability continue to improve, MO NCs obtained via continuous growth method are positioned to become enabling building blocks for optoelectronics, catalysis, and energy technologies. Looking ahead, several opportunities define a near-term roadmap. Predictive synthesis and operando control can be advanced by integrating continuous injection with *in situ* UV–vis absorption, small- and wide-angle X-ray scattering (SAXS/WAXS), or X-ray absorption spectroscopy (XAS), providing real-time signatures of nanocrystal size, faceting, and dopant activation.^230–233^ Machine-learning-assisted feedback could dynamically adjust injection rate, precursor identity, and temperature, enabling closed-loop growth that maintains target architectures.^234,235^

Looking forward, application spaces may broaden as multifunctional nanosystems mature. In the context of chromatic-tunable devices, for instance, radial dopant engineering is particularly attractive for dual-responsive materials that combine photochromic and electrochromic behavior within a single platform. Core-doped and shell-undoped structures can be designed so photoexcited carriers are preferentially driven toward, or away from, the surface, tuning the balance between light-driven coloration and electrically driven switching.^35,198,216^ This radial control can decouple, or intentionally couple, photochromic and electrochromic pathways. A given nanocrystal ensemble can show rapid, reversible electrochromic modulation under bias, while also exhibiting light-triggered coloration or bleaching driven by photoinduced charge redistribution. Such dual-responsive chromic nanocrystals could be integrated into smart windows, displays, and sensors where the optical state is switched by either an applied bias or illumination, and where electrical and optical stimuli can be combined to tune the magnitude and kinetics of the chromic response. The same design logic extends to photo–energy storage, where the aim is not only at harvesting photons but also at storing their photoinduced charges for later use. Dopant and charge localization can stabilize long-lived charge-separated states by spatially isolating electrons and holes in different regions of a nanocrystal thanks to the built-in electric field, or by creating radial band edges that trap one carrier type.^159,170,196,213^ For example, an electron-rich, heavily doped core combined with a more insulating shell can operate as a nanoscale charge reservoir, enabling persistent photocoloration and long-term storage of photogenerated electrons that can later be extracted electrochemically or released on demand. Embedding such nanocrystals into solid-state architectures could lead to photorechargeable electrochromic devices or integrated “color + energy” pixels where the optical state encodes stored charge. Finally, dopant profiles offer a practical design parameter for “dark photocatalytic” systems, in which catalytic activity persists well after illumination is switched off. By engineering nanocrystals to accumulate and stabilize photogenerated charges at specific radial positions (e.g., electrons near catalytically active surface sites while compensating holes are buried or neutralized), it becomes possible to create materials that are photocharged and remain catalytically active in the dark.^209,210^ In this sense, radially doped MO NCs bridge conventional photocatalysts, electrochemical catalysts, and charge-storage materials, suggesting a new generation of optoelectronic platforms where nanometer-scale architecture determines when, how, and under which stimulus photoelectrocatalytic driven functions are expressed.

## Vocabulary

doped metal oxide nanocrystals: metal-oxide nanoparticles engineered with dopants or defects that increase charge-carrier density, enabling tunable conductivity and plasmonic-like absorption
slow injection synthesis: a colloidal synthesis method where precursors are gradually added into the solvent, yielding control over size and dopant incorporation in metal oxide nanocrystals
dopant placement: spatial control over dopant atoms incorporation (both substitutional or interstitial) throughout the final nanocrystal structure from the core to the surface, allowing to engineer the charge carrier distribution and the resulting optoelectronic properties of the material
localized surface plasmon resonance: light-driven collective oscillation of free carriers confined in a nanoparticle resulting in an intense optical feature in an absorbance/extinction spectrum
depletion layer: a near-surface region of a doped metal oxide nanocrystal with reduced free-carrier density, whose controlled thickness and surface chemistry can affect the nanocrystal optoelectronic response
photodoping: light-induced accumulation of long-lived carriers in a metal oxide nanocrystal, enabled by a sacrificial redox partner, which raises the effective doping level and tunes its optical and electronic properties
